# Pyroptosis of pulmonary fibroblasts and macrophages through NLRC4 inflammasome leads to acute respiratory failure

**DOI:** 10.1016/j.celrep.2025.115479

**Published:** 2025-03-29

**Authors:** Yan Zhang, Guoying Zhang, Brittany Dong, Ankit Pandeya, Jian Cui, Samuel dos Santos Valenca, Ling Yang, Jiaqian Qi, Zhuodong Chai, Congqing Wu, Daniel Kirchhofer, Toshihiko Shiroishi, Fadi Khasawneh, Min Tao, Feng Shao, Christopher M. Waters, Yinan Wei, Zhenyu Li

**Affiliations:** 1 Department of Pharmaceutical Sciences, Texas A&M University, College Station, TX 77843, USA; 2 Department of Physiology, University of Kentucky, Lexington, KY 40506, USA; 3 Department of Chemistry, University of Kentucky, Lexington, KY 40506, USA; 4 Saha Cardiovascular Research Center, University of Kentucky, Lexington, KY 40506, USA; 5 Department of Surgery, University of Kentucky, Lexington, KY 40506, USA; 6 Department of Early Discovery Biochemistry, Genentech, South San Francisco, CA 94080, USA; 7 RIKEN BioResource Research Center, Tsukuba, Ibaraki 305-0074, Japan; 8 Department of Oncology, First Affiliated Hospital of Soochow University, Suzhou 215006, China; 9 National Institute of Biological Sciences, Beijing 102206 China; 10 Lead contact

## Abstract

The NAIP/NLRC4 inflammasome plays a pivotal role in the defense against bacterial infections, with its *in vivo* physiological function primarily recognized as driving inflammation in immune cells. Acute lung injury (ALI) is a leading cause of mortality in sepsis. In this study, we identify that the NAIP/NLRC4 inflammasome is highly expressed in both macrophages and pulmonary fibroblasts and that pyroptosis of these cells plays a critical role in lung injury. Mice challenged with gram-negative bacteria or flagellin developed lethal lung injury, characterized by reduced blood oxygen saturation, disrupted lung barrier function, and escalated inflammation. Flagellin-induced lung injury was protected in caspase-1 or GSDMD-deficient mice. These findings enhance our understanding of the NAIP/NLRC4 inflammasome’s (patho)physiological function and highlight the significant role of inflammasome activation and pyroptosis in ALI during sepsis.

## In brief

Zhang et al. demonstrate that activation of the NAIP/NLRC4 inflammasome leads to acute lung injury. They identify pyroptosis of pulmonary macrophages and fibroblasts as an important mechanism driving inflammasome-mediated lung damage.

## Graphical abstract

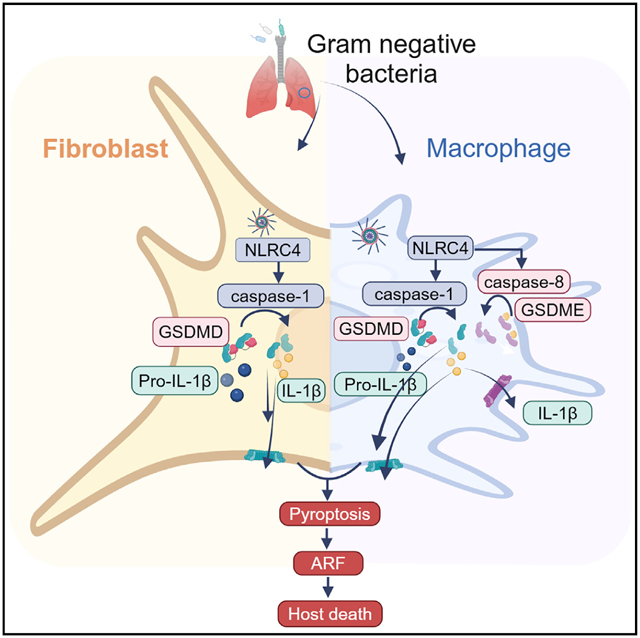

## INTRODUCTION

Activation of the NLRC4 inflammasome pathway stands as a critical defense mechanism against invasive pathogens.^1^ Nonetheless, an exaggerated inflammasome response can also precipitate a cytokine storm, coagulopathy, and tissue injury, and ultimately results in the demise of the host.^2^ Flagellins and conserved components of the type III secretion system (T3SS), the needle and rod proteins, of gram-negative bacteria are potent activators of NLRC4, which in turn trigger caspase-1 activation, leading to interleukin (IL)-1β and IL-18 maturation and release, along with initiating lytic cell death known as pyroptosis.^3–6^ Injection of either flagellins or the T3SS rod components into mice induces mortality in an NLRC4 inflammasome-dependent manner.^7^

The major function of the NAIP/NLRC4 inflammasome *in vivo* is to defend the host against bacterial infections, particularly those caused by bacteria possessing flagella and/or a T3SS, through the induction of inflammation involving the production of IL-1β and IL-18. Activation of the NAIP/NLRC4 inflammasome serves to reduce the growth of *Legionella pneumophila* growth in infected macrophages, as well as in the lungs and lymph nodes of infected mice,^8–12^ thereby safeguarding the host from lethal *L. pneumophila* infection.^13^ Furthermore, the activation of NLRC4 by flagellin has been shown to aid in the elimination of other bacterial strains, including *Listeria monocytogenes*,^14^ *Salmonella typhimurium*,^15–17^ and *Pseudomonas aeruginosa*.^18^ The (patho)physiological roles attributed to the NAIP/NLRC4 inflammasome *in vivo* are predominantly due to its activation within macrophages. Gain-of-function mutations of NLRC4 lead to spontaneous inflammasome activation, giving rise to pediatric enteritis and recurrent macrophage activation syndrome (MAS).^19,20^ Notably, our recent research has unveiled that NLRC4 activation by the rod protein of the *Escherichia coli* T3SS triggers disseminated intravascular coagulation (DIC), contributing to host mortality subsequent to caspase-1 activation and pyroptosis.^21^ In alignment with these findings, patients with NLRC4-MAS also exhibit coagulation abnormalities.^22^

Acute lung injury (ALI), along with its more severe manifestation, acute respiratory failure (ARF), is a leading cause of mortality in patients with sepsis, including those severely affected by COVID-19.^23,24^ ARF is a heterogeneous syndrome characterized by increased permeability of pulmonary capillary endothelial cells and dysfunction of epithelial surfaces.^25^ ALI is accompanied by inflammatory infiltrates into the lungs, including neutrophils and monocytes, and increases in proinflammatory cytokines.^26^ Nonetheless, the precise mechanism through which inflammation contributes to lung injury in the context of sepsis remains elusive.

Here, we observe that mice challenged with bacterial flagellin develop acute lethal respiratory failure. Flagellin-induced ARF is protected by deficiency of NAIPs, NLRC4, caspase-1, or Gasdermin D (GSDMD). We identify that lung fibroblasts express all essential components of the NAIP/NLRC4/caspase-1 inflammasome. Remarkably, flagellin-induced ARF is also ameliorated in fibroblast-specific GSDMD-deficient mice when macrophages are depleted. Our findings elucidate a molecular mechanism of ARF in sepsis and contribute to an enhanced understanding of the (patho)physiological role of the NAIP/NLRC4 inflammasome.

## RESULTS

### Bacterial flagellin causes ALI in mice

**Figure 1.**
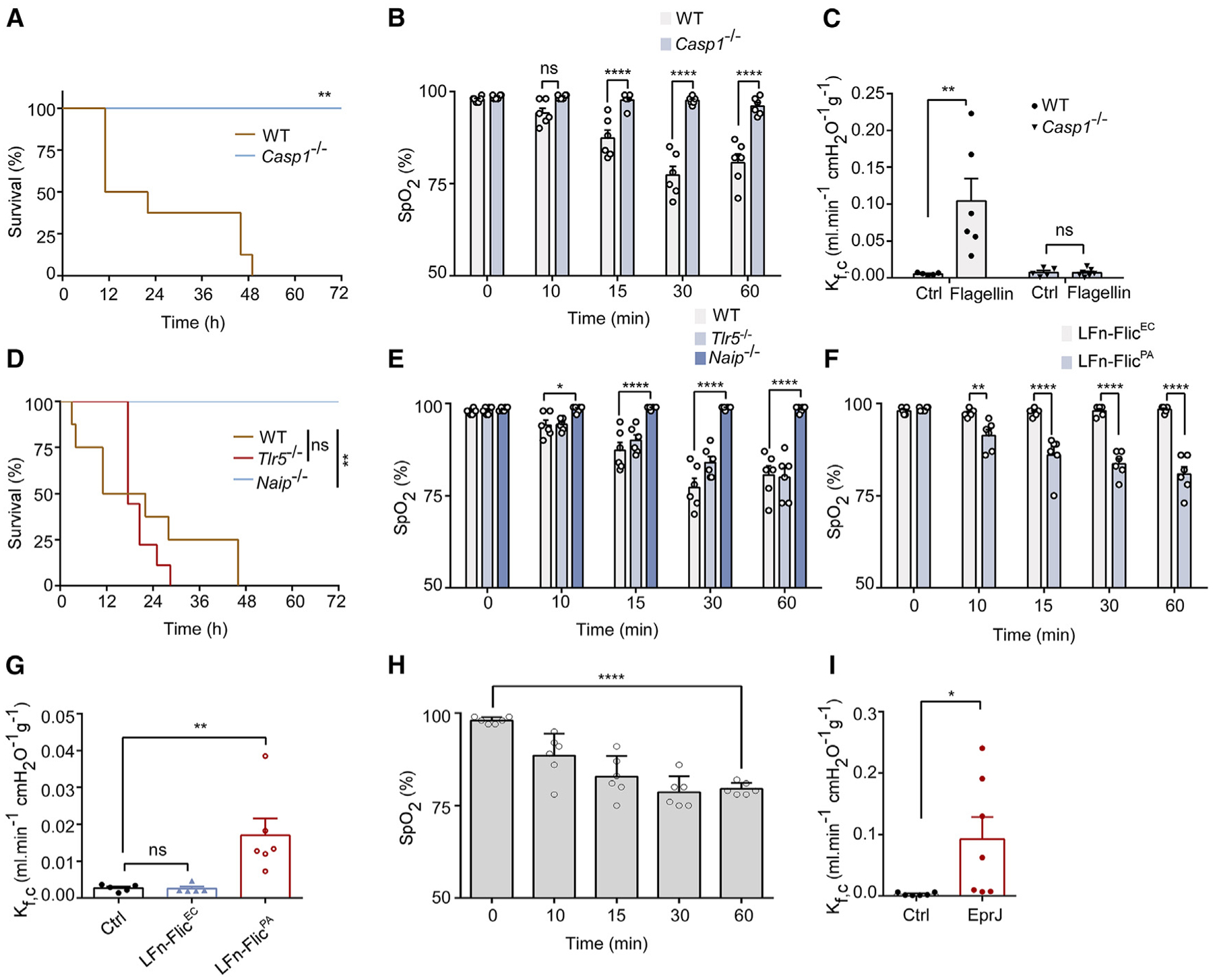
Flagellin-induced acute lung injury requires NAIP/NLRC4 inflammasome (A and B) Kaplan-Meier survival plots (A) and monitoring of arterial oxygen saturation (B) were conducted in WT or *Casp1*^−/−^ mice intravenously injected with 5 μg LFn-flagellin/PA. (C) Lung capillary filtration coefficient (K_f,c_) was measured before or 1 h after flagellin administration. (D and E) Kaplan-Meier survival plots for WT mice, *TLR5*^−/−^, or *NAIP*^−/−^-deficient mice challenged with 5 μg LFn-flagellin/PA (D), and monitoring of arterial oxygen saturation using infrared pulse oximetry (E). (F and G) WT mice intravenously injected with 5 μg LFn-Flic^EC/PA^ or LFn-Flic^PA/PA^. Arterial oxygen saturation (F) and lung K_f,c_ were determined (G). (H and I) WT mice injected intravenously with 5 μg LFn-EprJ/PA, with determination of arterial oxygen saturation (H) and lung K_f,c_ (I). Error bars represent the mean ± SEM. *p < 0.05; **p < 0.01;****p < 0.0001; ns, not significant, by log rank (Mantel-Cox) test (A and D), two-tailed unpaired t test (I), one-way ANOVA (G and H), or two-way ANOVA with Holm-Sidak multiple comparisons test (B, C, E, and F).

ARF is a leading cause of morbidity and mortality in sepsis. We utilized *L. pneumophila* flagellin, a potent activator of the NAIP/NLRC4 inflammasome,^9^ to investigate whether inflammasome activation results in ALI. Flagellin was fused to the cytosolic translocation domain of anthrax lethal factor (LFn) to enable efficient cytosolic delivery. LFn binds to anthrax protein protective agent (PA), which delivers the LFn-flagellin fusion protein into cytoplasm through receptor-mediated endocytosis.^5,27^ Purified LFn-flagellin, but not the nonfunctional 3A mutant as previously described,^12^ combined with PA, induced robust production of p20 caspase-1 and caused pyroptosis in mouse primary bone marrow-derived macrophages (BMDMs) (Figures S1A and S1B). Injection of LFn-flagellin/PA led to mortality in wild-type (WT), but not in the caspase-1-deficient mice (Figure 1A). To determine whether injection of LFn-flagellin/PA induced ALI, we monitored peripheral capillary oxygen saturation (SpO_2_). The normal SpO_2_ level is between 95% and 100%, and SpO_2_ levels around ~70% are life threatening. Injection of 5 μg LFn-flagellin/PA triggered a rapid decline in SpO_2_ level in WT mice, which was abolished by caspase-1 deficiency (Figure 1B).

Disruption of the vascular barrier, along with the leakage of fluid and proteins into lungs, remain a predominant contributor to acute respiratory injury during sepsis.^28,29^ Therefore, we investigated whether inflammasome activation by flagellin induced an increase in vascular permeability by measuring the mouse lung capillary filtration coefficient (K_f,c_).^30,31^ Following injection of LFn-flagellin/PA, the K_f,c_ was significantly elevated in WT mice, but not in the caspase-1-deficient mice (Figure 1C). Deficiency of caspase-1 also reduced flagellin-induced inflammation, especially at a low dose (Figure S2). Collectively, these data indicate that injection of flagellin into mice induced acute respiratory injury through the inflammasome pathway.

### Flagellin-induced lung injury depends on the NAIP/NLRC4 inflammasome

Flagellin is recognized by both the Toll-like receptor 5 (TLR5)^32–35^ on the cell membrane and the intracellular receptor NAIP5/6^5,36^ to activate the NLRC4 inflammasome. Administration of LFn-flagellin/PA caused lethality in both WT and the TLR5-deficient mice but not the mice lacking NAIP1–6 (Figure 1D), indicating that inflammasome activation is responsible for flagellin-induced mortality. Accordingly, injection of LFn-flagellin/PA resulted in acute respiratory injury in both WT and the TLR5-deficient mice but not in the NAIP1–6-deficient mice (Figure 1E). It has been reported that while the flagellins from *S. typhimurium*, *Yersiniosis enterocolitica*, and *P. aeruginosa* could bind to NAIP5 and activate the NLRC4 inflammasome, those from *enteropathogenic E. coli*, *enterohaemorrhagic E. coli*, *Shigella flexneri*, and *Burkholderia thailandensis* could not.^5^ Accordingly, flagellin from *P. aeruginosa* (Flic^PA^), but not the flagellin from *enterohaemorrhagic E. coli* (Flic^EC^), reduced SpO_2_ and disrupted endothelial barrier function in WT mice (Figures 1F and 1G). These data confirmed that acute respiratory injury induced by flagellin occurs, at least in part, through the inflammasome pathway. Supporting this conclusion, injection of PA with a rod protein of *E. coli* T3SS LFn-EprJ, a potent activator of the NLRC4 inflammasome,^21^ reduced SpO_2_ (Figure 1H) and increased the K_f,c_ in WT mice (Figure 1I).

### Flagellin-induced lung injury depends on pyroptosis

**Figure 2.**
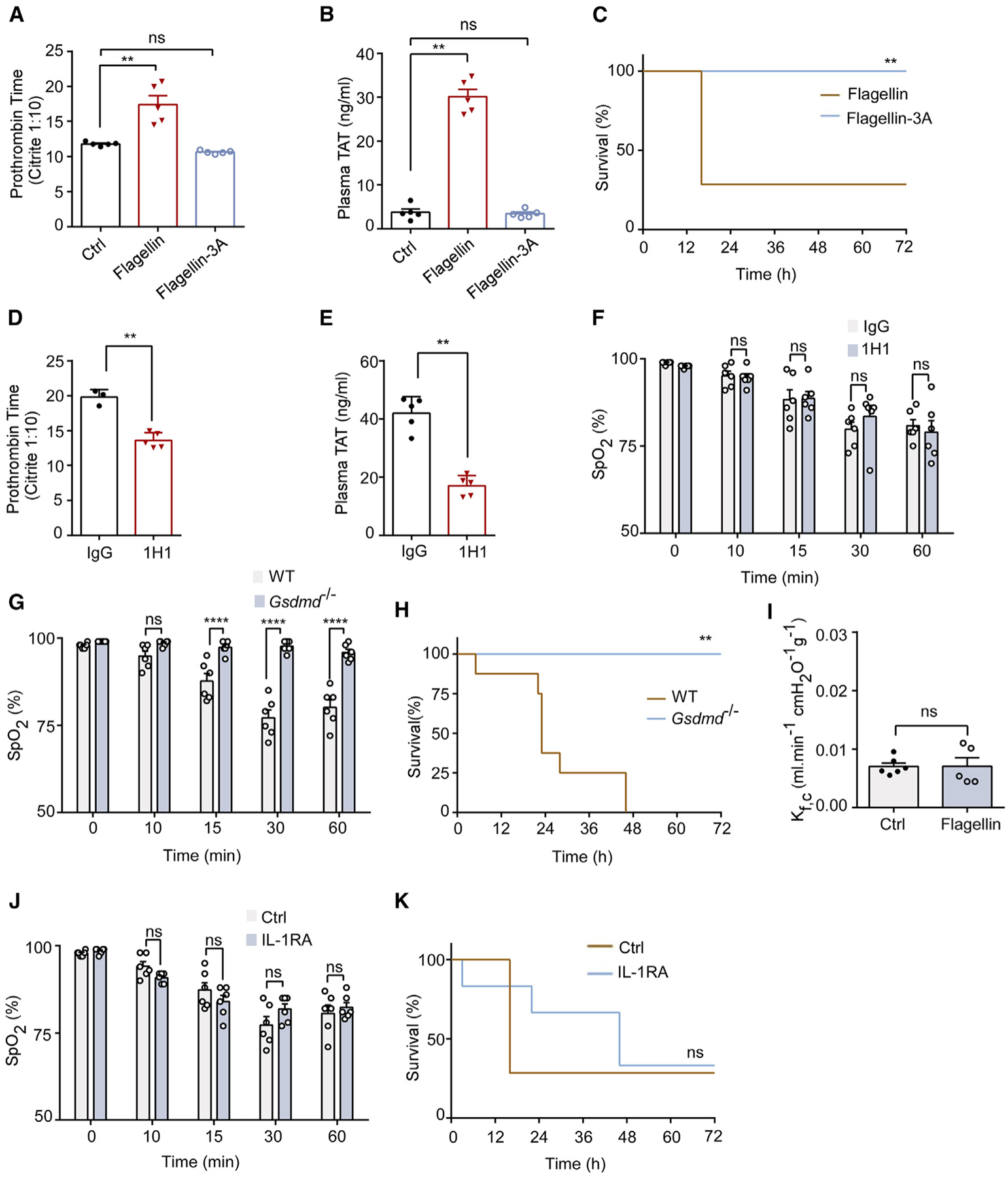
Lung cell pyroptosis plays a critical role in inflammasome-induced lung injury (A–C) WT mice received intravenous injections of PA (Ctrl), LFn-flagellin plus PA (3 μg of each per mouse), or the same dose of LFn-flagellin-3A mutant plus PA. Blood was collected 90 min post-injection. PT (A) and TAT (B) were measured. Kaplan-Meier survival plot is shown (C). (D–F) WT mice were injected intravenously with a rat immunoglobulin G or a rat anti-mouse TF-neutralizing antibody 1H1 (8 mg/kg). After 2 h, mice received flagellin, and blood was collected 90 min later. Prothrombin time (D), plasma TAT concentrations (E), and arterial oxygen saturation (F) were measured. (G–I) WT mice or *GSDMD*^−/−^ mice were injected intravenously with 5 μg LFn-flagellin/PA. Arterial oxygen saturation (G) and Kaplan-Meier survival plots are shown (H), and lung K_f,c_ was determined (I). (J and K) WT mice received intravenously IL-1RA (1 mg/kg body weight) 10 min prior to injection of LFn-flagellin/PA. Arterial oxygen saturation and Kaplan-Meier survival plots are shown. Error bars represent the mean ± SEM. **p < 0.01;****p < 0.0001; ns, not significant, by log rank (Mantel-Cox) test (C, H, and K), two-tailed unpaired t test (D, E, and I), one-way ANOVA (A and B), or two-way ANOVA with Holm-Sidak multiple comparisons test (F, G, and J).

We reported recently that inflammasome activation and subsequent pyroptosis induced by injection of the T3SS rod proteins of the gram-negative bacteria triggered DIC and lethality in mice.^21^ Tissue factor (TF) released from pyroptotic macrophages initiates coagulation and thrombosis. As expected, the injection of LFn-flagellin/PA, but not the flagellin 3A mutant, into WT mice also induced coagulopathy or DIC and lethality, as evidenced from prolonged plasma clotting time and increased plasma levels of thrombin-antithrombin III (TAT) complexes (Figures 2A–2C). DIC is believed to contribute to tissue damage during sepsis.^37^ Therefore, we investigated whether ARF induced by flagellin was due to DIC. WT mice were pre-treated with a rat anti-mouse TF antibody, 1H1,^21^ prior to the injection of LFn-flagellin/PA. Although pre-treatment of the mice with 1H1 prevented flagellin-induced DIC (Figures 2D and 2E), it had no effect on blood SpO_2_ levels (Figure 2F), indicating that flagellin-induced acute respiratory injury is independent of DIC and thrombosis.

Direct consequences of inflammasome activation include the release of cytokines IL-1β and IL-18, as well as pyroptosis.^6,38,39^ Next, we used GSDMD-deficient mice to determine whether ARF following inflammasome activation is due to pyroptosis. GSDMD deficiency protected against flagellin-induced lethality, ALI, and disruption of the endothelial barrier function (Figures 2G–2I). GSDMD deficiency also reduced flagellin-induced inflammation, especially at a low dose (Figure S2). However, pretreatment of mice with a specific IL-1 receptor antagonist, anakinra,^40^ had no effect on flagellin-induced ARF (Figures 2J and 2K). These findings suggest that pyroptosis following inflammasome activation is responsible for flagellin-induced ARF.

### Flagellin-induced lung injury extends beyond macrophage pyroptosis

**Figure 3.**
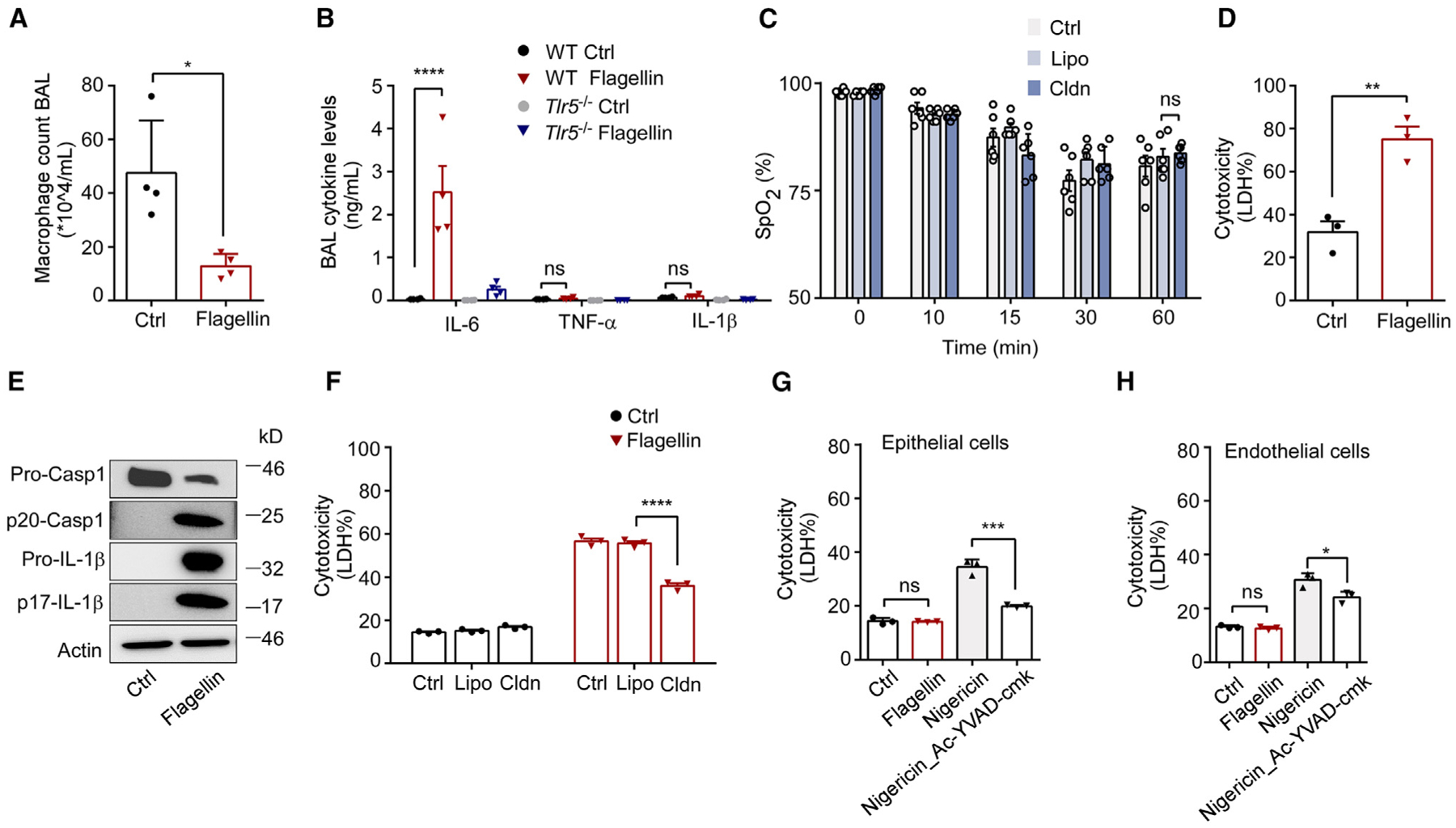
Flagellin-induced lung injury extends beyond macrophage pyroptosis (A and B) WT or *TLR5*^−/−^ mice were intravenously injected with 5 μg LFn-flagellin/PA. After 1 h, the total number of macrophages (A) and cytokine levels (B) in BAL fluid were measured. (C) WT mice received PBS, control liposomes (Lipo), or clodronate-containing liposomes (Cldn) 24 h prior to injection of 5 μg LFn-flagellin/PA. Arterial oxygen saturation was monitored. (D and E) Lung cells were incubated with PA (Ctrl) or 1 μg/mL LFn-flagellin/PA for 6 h. LDH concentration (D) and levels of p20 caspase-1 and p17 IL-1β by immunoblotting (E) were detected. (F) Lung cells were pre-incubated with PBS, 100 μg/mL Lipo, or Cldn, then transfected with PA (Ctrl) or 1 μg/mL LFn-flagellin/PA for 6 h. LDH concentrations were measured. (G and H) Epithelial (G) and endothelial (H) cells were incubated with PA (Ctrl) or 1 μg/mL LFn-flagellin/PA for 6 h. As a positive control, cells were primed with 1 μg/mL LPS for 4 h, followed by stimulation with 20 μM nigericin for 4 h, with or without caspase-1 inhibitor Ac-YVAD-cmk (5 μM). LDH concentrations in the supernatant were measured to determine cytotoxicity. Error bars represent the mean ± SEM. *p < 0.05; **p < 0.01; ***p < 0.001; ****p < 0.0001; ns, not significant, by two-tailed unpaired t test (A and D), one-way ANOVA (G and H), or two-way ANOVA with Holm-Sidak multiple comparisons test (B, C, and F).

Macrophages are the major resident immune cells in lungs.^41^ We first examined whether macrophage pyroptosis contributes to ALI following NLRC4 activation. We measured cytokine and macrophage cell concentrations in the bronchoalveolar lavage (BAL) fluid obtained from mice challenged with LFn-flagellin/PA. As expected, macrophages concentrations were significantly lower after the challenge with flagellin (Figures 3A and S3A), which is likely due to pyroptosis (Figure S3B). To determine whether treatment with LFn-flagellin/PA could induce inflammation, we measured tumor necrosis factor α, IL-1β, and IL-6 concentrations in BAL and found that only IL-6 concentration was increased (Figure 3B). Injection of LFn-flagellin/PA also induced leukocyte recruitment into the lungs in WT mice, but much less in the caspase-1- or GSDMD-deficient mice (Figure S2).

To determine whether macrophage pyroptosis contributes to flagellin-induced ARF, WT mice were treated with intravenous clodronate to deplete monocytes and macrophages prior to flagellin challenge. Clodronate administration depleted monocytes from circulation (>95%)^42^ and also significantly reduced resident lung macrophages (~67%) (Figure S3C). However, pre-depletion of monocytes/macrophages did not significantly improve the reduction in blood SpO_2_ levels after flagellin challenge (Figure 3C). While these data do not exclude the potential involvement of macrophage pyroptosis in flagellin-induced ARF, they strongly imply that other cell types might also be undergoing pyroptosis, which in turn contributes to flagellin-induced ALI and declines in SpO_2_ levels following inflammation activation.

Next, we used *in vitro* experiments to identify the cell types that may undergo pyroptosis following inflammasome activation. As expected, incubation of a suspension of total mouse lung cells with LFn-flagellin/PA induced pyroptosis as revealed by the lactate dehydrogenase (LDH) release assay, and caspase-1 activation, evidenced by caspase-1 and IL-1β cleavage (Figures 3D and 3E). Depletion of macrophages by incubation of the total lung cells isolated from WT mice with clodronate reduced but did not abolish LFn-flagellin/PA-induced cell death (Figures 3F and S3D). Lung epithelial cells, including type I and type II alveolar cells, fibroblasts, and endothelial cells, are the major structural cell types in the alveolar tissue.^43^ Epithelial cells and endothelial cells form the alveolar-capillary membrane where gas exchange occurs. Therefore, we hypothesized that pyroptosis may occur in epithelial cells and/or endothelial cells leading to acute respiratory injury following inflammasome activation. To test this hypothesis, we isolated epithelial cells and endothelial cells from mouse lungs and incubated them with LFn-flagellin/PA. Surprisingly, pyroptosis did not occur in either epithelial cells or endothelial cells after incubation with LFn-flagellin/PA, as demonstrated by the lack of LDH release and negative propidium iodide (PI) staining (Figures 3G, 3H, and S3E). These results were verified using commercial mouse epithelial cells and endothelial cells (Figures S4A and S4B). In contrast, nigericin, an activator of the NLRP3 inflammasome, triggered pyroptosis of epithelial cells or endothelial cells (Figures 3G and 3H).

### Pulmonary fibroblasts undergo pyroptosis following NLRC4 inflammasome activation, contributing to flagellin-induced lung injury

**Figure 4.**
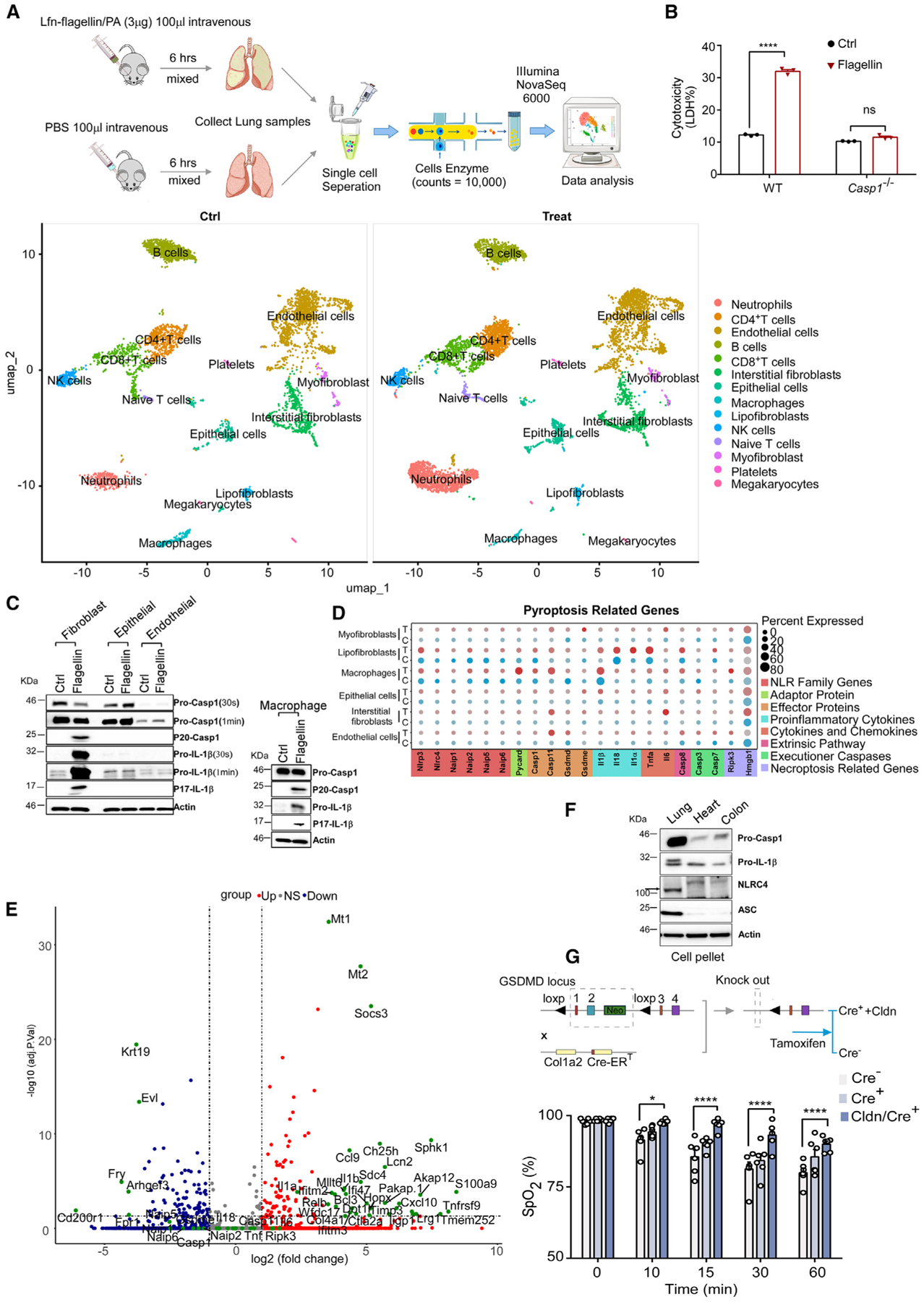
Pulmonary fibroblast is responsible for flagellin-induced lung injury (A) The procedure of scRNA-seq and uniform manifold approximation and projection plot displaying distinct changes in different cell types within the lungs following challenge with LFn-flagellin/PA. (B) Fibroblasts isolated from WT mice and *Casp1*^−/−^ mice were incubated with PA (Ctrl) or 1 μg/mL LFn-flagellin/PA for 6 h. Cytotoxicity was determined by LDH assay. (C) Bone marrow-derived macrophages, as well as lung epithelial, endothelial, and fibroblasts isolated from untreated WT mice were incubated with PA (Ctrl) or 1 μg/mL LFn-flagellin/PA for 6 h. P20 caspase-1 and p17 IL-1β were detected by immunoblot. (D and E) Significant changes in gene expression in the different cell populations (D), including lipofibroblast (E) population following flagellin treatment. (F) NLRC4 inflammasome components were detected in fibroblasts from lung, heart, and colon tissues of mice. (G) *GSDMD*^*fl/fl*^*/Col1a2*^*Cre*−^ or *GSDMD*^*fl/fl*^*/Col1a2*^*Cre+*^ mice were injected intravenously with 5 μg LFn-flagellin/PA. Cre^+^ mice were further treated with Cldn 24 h prior to flagellin injection. Arterial oxygen saturation was monitored using infrared pulse oximetry. Error bars represent the mean ± SEM. *p < 0.05; ****p < 0.0001; ns, not significant, by two-way ANOVA with Holm-Sidak multiple comparisons test (B and G).

To identify the types of cells that are responsible for inflammasome-dependent ALI, we performed single-cell RNA sequencing (scRNA-seq) of lung cells. Three mice were injected with PBS or LFn-flagellin/PA. After 90 min, cells were isolated and pooled from the lungs of the three mice in each group and were subjected to RNA-seq. Following the challenge with LFn-flagellin/PA, the total immune cells increased dramatically in mouse lungs (Figures 4A and S4C; Table 1). This is mainly due to increases in neutrophil and T cells in the lungs (Figure S4C; Table 1). As expected, the population of macrophages in lungs significantly decreased after the challenge with LFn-flagellin/PA (Figure S4C and Table 1), likely due to pyroptosis. In addition to these cell types, fibroblasts and endothelial cells are the two major cell types that show a trend toward decreasing (Figures 4A and S4C). The reduction in the endothelial cell population aligns with the impairment of endothelial barrier function in mice challenged with LFn-flagellin/PA (Figure 1C). However, since LFn-flagellin/PA did not directly induce pyroptosis in endothelial cells, it is likely that death of endothelial cells *in vivo* is caused indirectly by a mediator(s) released from pyroptosis of other types of cells.

It has been reported recently that pulmonary fibroblasts play an essential role in ALI induced by respiratory viral infection.^44^ Consistent with the scRNA-seq results, we observed that pyroptosis occurred in pulmonary fibroblasts following incubation with LFn-flagellin/PA in WT mice, which was abolished in the caspase-1-deficient mice (Figures 4B and S3E). Moreover, treatment with LFn-flagellin/PA specifically activated caspase-1 and led to IL-1β cleavage in mouse pulmonary fibroblasts, but not in epithelial cells or endothelial cells (Figure 4C). Consistent with these findings, data from the scRNA-seq indicate that pulmonary fibroblasts express all the components of the NLRC4 inflammasome, including NAIP5, NLRC4, and caspase-1 (Figures 4D and 4E). In contrast, epithelial and endothelial cells do not express high levels of these inflammasome components (Figures S4D and S4E). We further confirmed that pulmonary fibroblasts express all the components of the NLRC4 inflammasome, including ASC, NLRC4, and caspase-1 by western blot (Figure 4F). We also isolated fibroblasts from different tissues, including intestine and heart, and compared the expression of the inflammasome proteins in those fibroblasts. Interestingly, only the pulmonary fibroblasts but not the fibroblasts from heart and intestine exhibited high levels of ASC, NLRC4, and caspase-1 (Figure 4F).

To determine whether pyroptosis of pulmonary fibroblasts contributes to ALI induced by flagellin, we generated fibroblast-specific GSDMD-deficient mice by crossbreeding the GSDMD flox mice with the Col1a2 Cre-ER mutant mouse strain. Reduction of GSDMD expression was induced by administering tamoxifen and verified by western blot (Figure S5A). Reduction in SpO_2_ by injection of LFn-flagellin/PA was slightly protected in the fibroblast-specific GSDMD-deficient mice but did not reach statistical significance (Figure 4G). However, when macrophages were pre-depleted, the fibroblast-specific GSDMD-deficient mice showed significant protection against the lung damage induced by LFn-flagellin/PA (Figures 4G, S5B, and S5C). These data suggest that pyroptosis of both fibroblasts and macrophages contributes to the development of ALI.

### ALI induced by high-dose flagellin involves macrophage pyroptosis through the caspase-8/GSDME pathway

**Figure 5.**
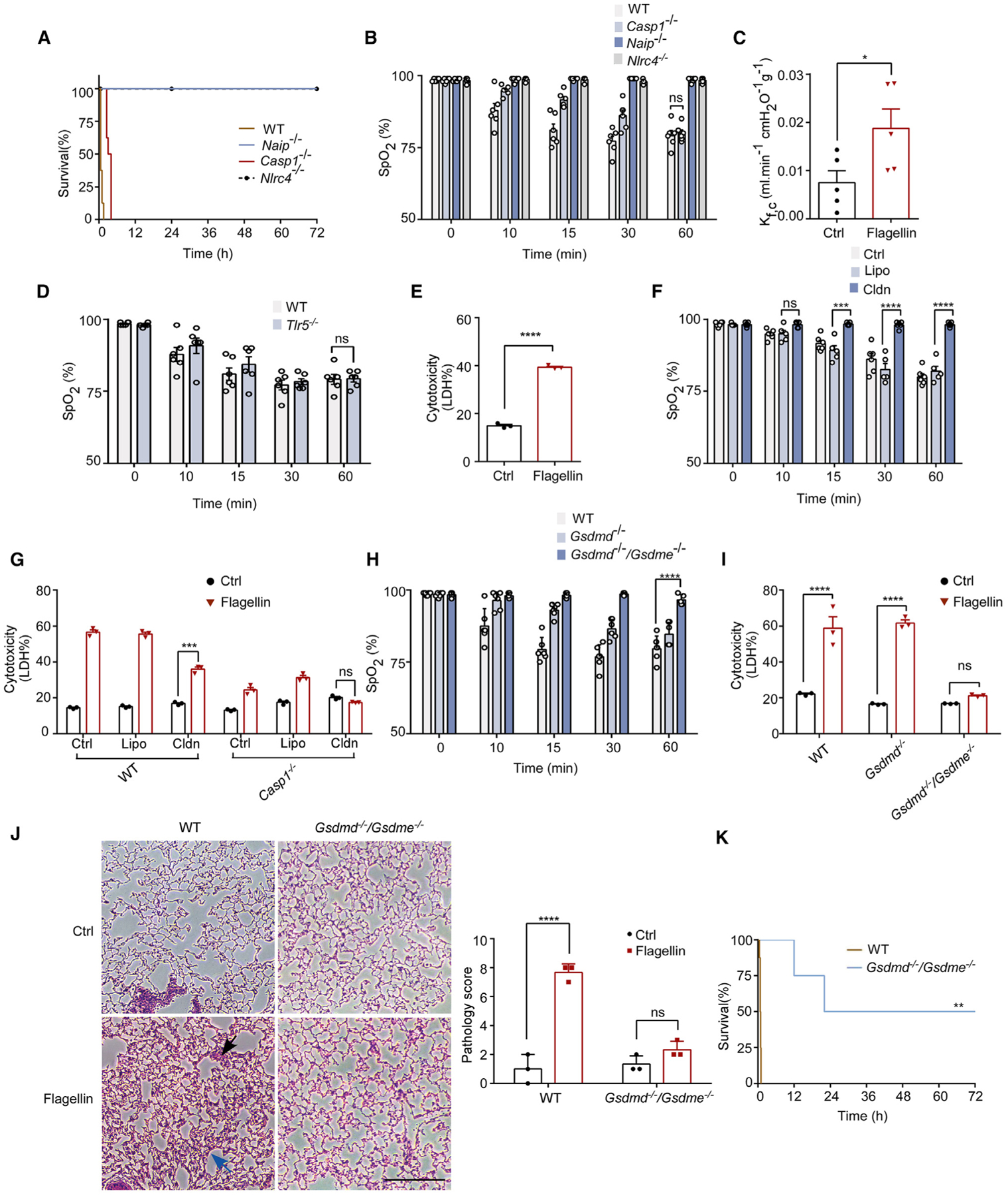
Caspase-8/GSDME pathway in macrophage contributed to acute lung injury induced by high-dose flagellin (A and B) WT mice, *Casp1*^−/−^, *NAIP*^−/−^, or *Nlrc4*^−/−^ mice were injected with 10 μg LFn-flagellin/PA. Kaplan-Meier survival plots (A) and arterial oxygen saturation were monitored (B). (C) *Casp1*^−/−^ mice received 10 μg LFn-flagellin/PA. The lung K_f,c_ was determined. (D) WT mice or *TLR5*^−/−^-deficient mice received 10 μg LFn-flagellin/PA. Arterial oxygen saturation was monitored. (E) Total lung cells isolated from *Casp1*^−/−^ mice were incubated with PA (Ctrl) or 10 μg/mL LFn-flagellin/PA for 6 h. Cytotoxicity was determined by LDH assay. (F) *Casp1*^−/−^ mice were pre-treated with PBS, Lipo, or Cldn 24 h prior to injection of 10 μg LFn-flagellin/PA. Arterial oxygen saturation was monitored. (G) Total lung cells isolated from WT mice or *Casp1*^−/−^ mice were pre-incubated with PBS and 100 μg/mL Lipo or Cldn, and then incubated with PA or 1 μg/mL LFn-flagellin/PA for 6 h. LDH in the supernatant was measured. (H) WT, *GSDMD*^−/−^, and *GSDMD*^−/−^*/GSDME*^−/−^ mice were injected 10 μg LFn-flagellin/PA. Arterial oxygen saturation was monitored. (I) Total lung cells isolated from WT, *GSDMD*^−/−^, and *GSDMD*^−/−^*/GSDME*^−/−^ mice were incubated with PA or 1 μg/mL LFn-flagellin/PA for 6 h. LDH in supernatant was measured. (J and K) WT and *GSDMD*^−/−^*/GSDME*^−/−^ mice were injected 10 μg LFn-flagellin/PA. Histological evaluation of lung morphology (J) and Kaplan-Meier survival plots (K) were measured. Black arrow points to mononuclear cell infiltration in lungs, blue arrow points to thickened alveolar wall, and red arrow points to hemorrhage. Scale bars, 200 μm. Error bars represent the mean ± SEM. *p < 0.05; **p < 0.01; ***p < 0.001; ****p < 0.0001; ns, not significant, by log rank (Mantel-Cox) test (A and K), two-tailed unpaired t test (C and E), or two-way ANOVA with Holm-Sidak multiple comparisons test (B, D, and F–I).

Although deficiency of caspase-1 protected against ALI and mortality following injection of 5 μg LFn-flagellin/PA (Figures 1A and 1B), administration of a higher dose of LFn-flagellin/PA (10 μg) killed the caspase-1-deficient mice, but not the mice lacking NAIP1–6 or NLRC4 (Figure 5A). To determine whether ALI is the major cause of death in the caspase-1-deficient mice challenged with high-dose LFn-flagellin/PA, we measured blood SpO_2_ levels in the caspase-1-deficient mice. Indeed, injection of 10 μg LFn-flagellin/PA rapidly reduced blood SpO_2_ levels and induced lung damage in both WT and the caspase-1 deficient mice (Figures 5B and S2). Accordingly, endothelial barrier function was disrupted in caspase-1-deficient mice (Figure 5C). Mice lacking NAIP1–6 and NLRC4 (Figure 5B) but not TLR5 (Figure 5D) were protected from acute respiratory injury under this condition. These data suggest that there exists a NLRC4-mediated but caspase-1-independent mechanism responsible for flagellin-induced acute respiratory injury. In support of this hypothesis, incubation of total lung cells from the caspase-1-deficient mice with 10 μg/mL LFn-flagellin/PA induced cell death (Figure 5E). However, incubation of pulmonary fibroblasts isolated from the caspase-1-deficient mice with 10 μg/mL LFn-flagellin/PA failed to induce cell death (Figure S6A). These data suggest that at high concentrations, flagellin could induce pyroptosis in cell types other than fibroblasts, which also contributes to flagellin-induced respiratory injury. We then investigated whether 10 μg/mL LFn-flagellin/PA could induce pyroptosis of lung epithelial cells or endothelial cells and observed no cell death (Figures S4A and S4B).

Flagellin could induce macrophage death through the caspase-8-dependent pathway^45^ (Figures S6B and S6C), which involves both apoptosis and Gasdermin E (GSDME)-dependent pyroptosis.^45^ Thus, we hypothesized that in the absence of caspase-1, flagellin might induce macrophage pyroptosis through caspase-8, thereby contributing to acute respiratory injury. To test this hypothesis, we pre-depleted macrophages with clodronate in the caspase-1-deficient mice, and then injected these mice with 10 μg LFn-flagellin/PA. Although pre-depletion of macrophages failed to prevent the reduction of SpO_2_ levels in WT mice (Figure 3C), it successfully prevented the decrease in SpO_2_ in the caspase-1-deficient mice (Figure 5F). Consistent with the *in vivo* results, pre-depletion of macrophages in caspase-1-deficient total lung cells abolished flagellin-induced cell death (Figure 5G). In contrast, flagellin-induced cell death was only partially inhibited by caspase-1 deficiency or macrophage depletion. Deficiency of both GSDMD and GSDME, rather than GSDMD alone, protected against ALI induced by the administration of 10 μg LFn-flagellin/PA (Figures 5H and S2). Accordingly, flagellin-induced cell death and lung damage in mouse lungs were abolished by deficiency of both GSDMD and GSDME (Figures 5I and 5J). Additionally, deficiency in both GSDMD and GSDME improved survival rates following flagellin exposure (Figure 5K). Collectively, these data indicate that apart from fibroblast pyroptosis, macrophage pyroptosis mediated by the caspase-8/GSDME pathway also contributes to ALI in response to high-dose flagellin challenge.

**Figure 6.**
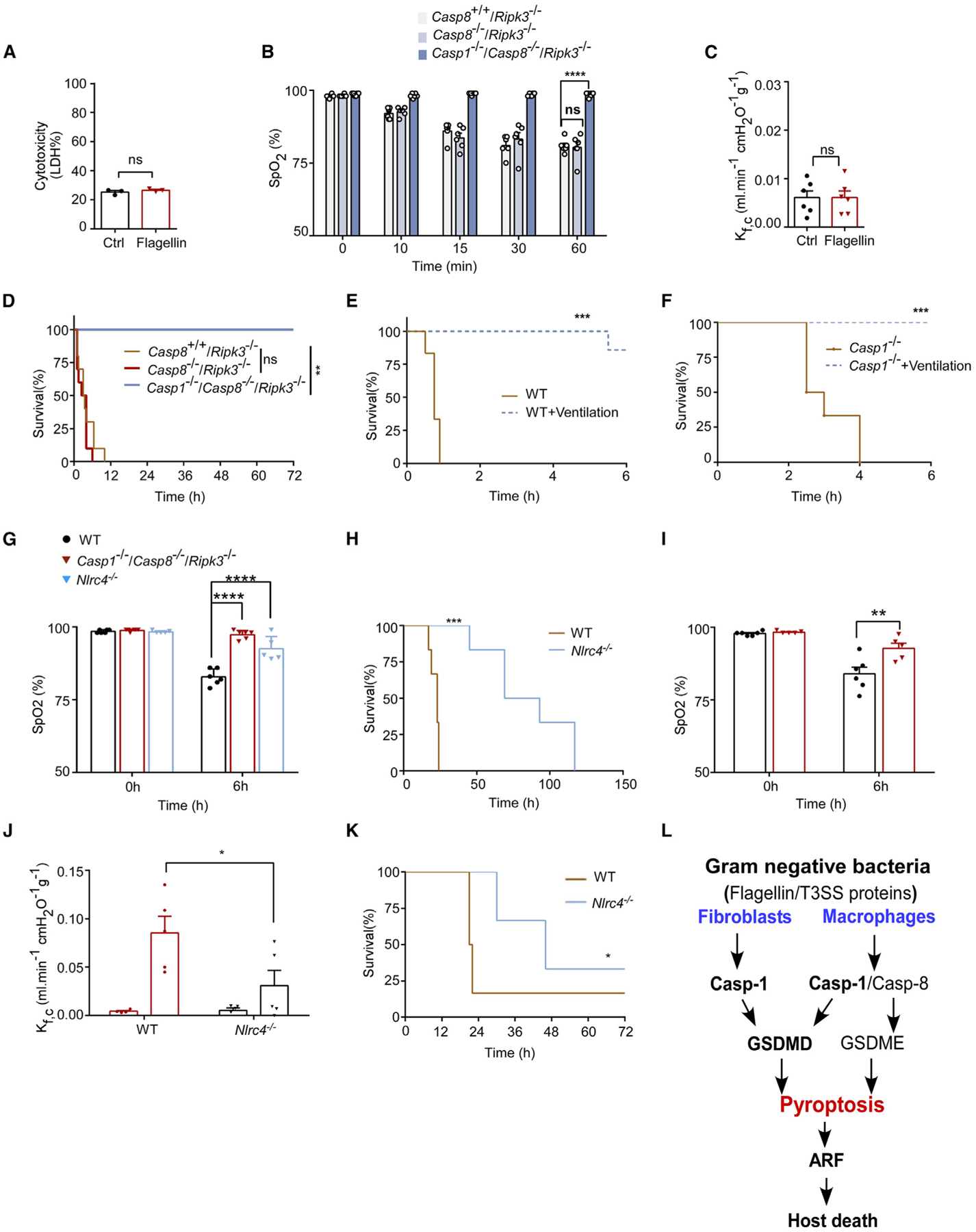
Caspase-8-dependent pyroptosis of macrophages contributes to lung injury in the absence of caspase-1 (A) Total lung cells isolated from WT and *Casp1*^−/−^*/Casp8*^−/−^*/Ripk3*^−/−^ mice were incubated with 1 μg/mL LFn-flagellin/PA for 6 h. Cell culture supernatants were used to measure LDH concentration. (B–D) *Ripk3*^−/−^mice, *Casp8*^−/−^*/Ripk3*^−/−^ mice, and *Casp1*^−/−^*/Casp8*^−/−^*/Ripk3*^−/−^ mice received 10 μg LFn-flagellin/PA. Arterial oxygen saturation (B), lung K_f,c_ (C), and Kaplan-Meier survival plots (D) for flagellin-challenged mice are shown. (E and F) WT (E) and *Casp1*^−/−^ mice (F) underwent mechanical ventilation and were injected intravenously with 10 μg LFn-flagellin/PA for up to 6 h. Kaplan-Meier survival plots for mice challenged with flagellin are shown. (G) WT, *Nlrc4*^−/−^, and *Casp1*^−/−^*/Casp8*^−/−^*/Ripk3*^−/−^ mice were injected intraperitoneally with 2 × 10^8^ CFU *Salmonella typhimurium* for 6 h. Arterial oxygen saturation was measured. (H) WT and *Nlrc4*^−/−^ mice were injected intraperitoneally with 2 × 10^8^ CFU *S. typhimurium*. Kaplan-Meier survival plots are shown. (I–K) WT and *Nlrc4*^−/−^ mice were subjected to the FIP model. Arterial oxygen saturation (I), lung K_f,c_ (J), and Kaplan-Meier survival analysis (K) following administration of a 40-mg/mL feces solution are shown. (L) Model of lung injury triggered by caspase-1-dependent and -independent inflammasome activation. Error bars represent the mean ± SEM. *p < 0.05; **p < 0.01; ***p < 0.01; ****p < 0.0001; ns, not significant, by log rank (Mantel-Cox) test (D–F, H, and K), two-tailed unpaired t test (A and C), or two-way ANOVA with Holm-Sidak multiple comparisons test (B, G, I, and J).

If this hypothesis is true, then deficiency of both caspase-1 and caspase-8 should abolish flagellin-induced cell death in total lung cells *in vitro*, along with mitigating acute respiratory injury in response to the challenge of 10 μg LFn-flagellin/PA *in vivo*. This hypothesis was tested using *Casp1*^−/−^*/Casp8*^−/−^*/Ripk3*^−/−^ mice, generated by crossbreeding *Casp8*^−/−^*/Ripk3*^−/−^ mice^46^ with *Casp1*^−/−^ mice. *In vitro* experiments revealed a complete absence of cell death induced by 10 μg LFn-flagellin/PA in total lung cells derived from *Casp1*^−/−^*/Casp8*^−/−^*/Ripk3*^−/−^ mice (Figure 6A). Consistent with the *in vitro* findings, ARF induced by 10 μg LFn-flagellin/PA did not manifest in *Casp1*^−/−^*/Casp8*^−/−^*/Ripk3*^−/−^ mice (Figure 6B), and disruption of the endothelial barrier function was not observed in *Casp1*^−/−^*/Casp8*^−/−^*/Ripk3*^−/−^ mice (Figure 6C). Upon administration of 10 μg LFnflagellin/PA, both WT mice and caspase-1-deficient mice died within 6 h, while *Casp1*^−/−^*/Casp8*^−/−^*/Ripk3*^−/−^ mice but not *Casp8*^−/−^*/Ripk3*^−/−^ mice survived (Figure 6D).

### Mechanical ventilation with positive pressure prolongs survival of the mice challenged with flagellin

To determine whether the lethality associated with inflammasome activation was attributed to acute respiratory injury, mice were subjected to an injection with 10 μg LFn-flagellin/PA, followed by intubation and subsequent positive pressure mechanical ventilation. While WT mice died within 1 h and *Casp1*^−/−^ mice died within 4 h, mechanical ventilation extended survival for up to 6 h for both WT (except one) and *Casp1*^−/−^ mice (Figures 6E and 6F). These data indicate that mortality linked to inflammasome activation stems from ARF. Next, we investigated the relevance of our findings in the context of bacterial infection using a mouse model of acute septic shock induced by *Salmonella*. Notably, we observed that the reduction in SpO_2_ was mitigated in *Casp1*^−/−^*/Casp8*^−/−^*/Ripk3*^−/−^ mice, as well as *Nlrc4*^−/−^ mice (Figure 6G). Consequently, *Salmonella*-induced lung damage and survival were improved in the NLRC4-deficient mice (Figures 6H and S6D). Moreover, using a fecal-induced peritonitis (FIP) sepsis model, we found that the NLRC4 deficiency largely protected against lung injury (Figures 6I and 6J) and increased survival rate (Figure 6K).

## DISCUSSION

Many gram-negative bacteria, including *S. typhimurium*, *L. pneumophila*, *P. aeruginosa*, *E. coli*, and *Shigella flexneri*, are able to activate the NAIP/NLRC4 inflammasome. The NAIP/NLRC4 inflammasome detects bacterial flagella as well as the T3SS rod and needle proteins of these strains. Until now, the primary role attributed to the NAIP/NLRC4 inflammasome has been its activation in triggering the inflammatory response of the host, thereby contributing to defense against infection by limiting bacterial proliferation within infected cells, predominantly macrophages. Our studies have revealed that the NAIP/NLRC4 inflammasome is highly expressed in pulmonary fibroblasts. We demonstrate that pyroptosis of pulmonary fibroblasts triggers ALI, leading to respiratory failure and host death. This discovery enhances our understanding of the role of the NAIP/NLRC4 inflammasome in infectious diseases.

Although activation of inflammatory caspases plays a key role in the innate immune response against bacterial infections, excessive activation of these caspases leads to DIC, multi-organ failure, and host lethality.^2,7,47^ In this study, we identified that inflammasome activation leads to ARF, a lethal complication of sepsis. Inflammasome activation promotes inflammation through the generation and subsequent release of cytokines such as IL-1β and IL-18, in addition to inducing pyroptosis.^6,38,39^ We demonstrate that GSDMD deficiency, but not the IL-1 receptor antagonist anakinra, protects against flagellin-respiratory failure. This finding suggests that pyroptosis, rather than IL-1 signaling, plays a major role in the ALI following inflammasome activation. Our data also indicate that flagellin can induce IL-6 production in a TLR5-dependent manner (Figure 3B). However, since TLR5 does not play a major role in flagellin-induced lung damage, it is reasonable to speculate that IL-6 does not play a significant role in flagellin-induced lung injury. Given that fibroblast-specific GSDMD depletion protected against flagellin-induced lung injury in the macrophage-depleted mice, it is plausible that pyroptosis of both fibroblasts and macrophages serves as the primary instigator of lung injury. The role of the endothelial barrier function in maintaining optimal lung function is well established. In the flagellin-challenged mice, we detected inflammation and disruption of the endothelial barrier function in lungs, suggesting that this secondary response might also contribute to lung injury. The data from scRNA-seq indicates that the concentration of lung endothelial cells was also diminished following flagellin challenge. However, treatment with flagellin/PA failed to trigger pyroptosis of lung endothelial cells *in vitro*. These data suggest that the demise of endothelial cells is likely an indirect consequence, mediated by *in vivo* factors originating from pyroptosis. The intricate mechanisms that lead to endothelial cell death and the subsequent disruption of endothelial barrier function following inflammasome activation by flagellin demand further exploration.

Although macrophage pre-depletion did not confer protection against flagellin-induced lung injury in WT mice, the role of macrophage pyroptosis in ALI remains undeniable. This is evident because full protection against flagellin-induced lung injury upon high-dose flagellin challenge requires a combination of pre-depletion of macrophages and deficiency of caspase-1 in fibroblasts. These findings indicate the existence of a caspase-1-independent pathway that orchestrates macrophage activation when confronted with high doses of flagellin. Previous studies have reported that flagellin could induce macrophage death through the caspase-8-dependent pathway.^45^ Consistent with these earlier observations, we found that high-dose flagellin-induced lung injury and lethality were completely abolished in the *Casp1*^−/−^*/Casp8*^−/−^*/Ripk3*^−/−^ mice. Since high-dose flagellin-induced lung injury was also mitigated in the GSDMD/E double-knockout mice, and given that pyroptosis of fibroblasts relies on the caspase-1/GSDMD pathway, these data imply that GSDME contributes to ALI by mediating macrophage pyroptosis. It is known that caspase-8 mediates macrophage apoptosis through caspase-3, which is independent of GSDME.^45^ Consistent with previous findings, we found that flagellin induced cell death of the BMDMs from GSDMD/GSDME double-knockout mice (Figure S6E). In contrast, flagellin failed to induce cell death of the lung cells from GSDMD/GSDME double-knockout mice. These data suggest that caspase-8-mediated pyroptosis rather than apoptosis contributes to death of lung cells and ALI following NLRC4 activation. While previous studies suggest that Ripk3-mediated necroptosis contributes to the pathogenesis of lipopolysaccharide (LPS)-induced acute respiratory distress syndrome^48,49^ and severe influenza infection,^50^ our data show that flagellin-induced pyroptosis and the subsequent lung injury were not protected by Ripk3 deficiency. These data suggest that Ripk3 does not play a major role in ALI following NLRC4 activation. However, we cannot rule out the potential contribution of Ripk3-mediated necroptosis to lung injury secondary to pyroptosis *in vivo*.

NLRC4 inflammasome activation induced by the injection of either flagellin or the T3SS proteins results in rapid host death within a few hours. This phenomenon seems to be attributed to ALI as a principal factor causing the swift lethality following NLRC4 inflammasome activation, as evidenced by the prolonged survival of flagellin-challenged mice when supplemented with O_2_ through positive pressure mechanical ventilation. Acute respiratory failure is a leading cause of mortality in patients with sepsis.^23,24^ In our study, we revealed a previously underestimated mechanism wherein the pyroptosis of pulmonary fibroblasts via the caspase-1 pathway and pyroptosis of macrophages through the caspase-1/8 pathway jointly contribute to the emergence of acute respiratory injury in septic conditions (Figure 6L).

### Limitations of the study

The work presented in this article is based on mouse sepsis models. In addition to pyrotpsis of macrophages and fibroblasts, other immune cells and nonimmune cells may play a role in lung damage during sepsis. Future studies will be necessary to elucidate the contribution from individual cell types to acute lung injury during sepsis. Moreover, development of appropriate assays for human samples from patients with sepsis may help better define the physiological importance and relevance of pyroptosis in sepsis.

## RESOURCE AVAILABILITY

### Lead contact

Further information and requests for resources and reagents should be directed to and will be fulfilled by the lead contact, Zhenyu Li (zli21@tamu.edu).

### Materials availability

This study did not generate new unique reagents.

### Data and code availability

- Data: scRNA-seq data have been deposited at GEO: GSE269196, are publicly available, and can be accessed at: https://www.ncbi.nlm.nih.gov/geo/query/acc.cgi?acc=GSE269196. The accession number is listed in the key resources table.
- Code: This paper does not report original code.
- Any additional information required to reanalyze the data reported in this paper is available from the lead contact upon request.

## STAR★METHODS

### EXPERIMENTAL MODEL AND STUDY PARTICIPANT DETAILS

#### Animals

Wild-type C57BL/6J, *Naip*^−/−^, *Tlr5*^−/−^, *Gsdme*^−/−^ and B6.Cg-Tg(Col1a2-cre/ERT,-ALPP)7Cpd/2J transgenic mice were purchased from the Jackson Laboratory. *Nlcr4*^−/−^, *Casp1*^−/−^ and *Casp8*^−/−^*/Ripk3*^−/−^ mice were from Genentech Inc. *Gsdmd*^−/−^ and GSDMD floxed mice were gifts from Toshihiko Shiroishi (RIKEN BioResource Research Center, Tsukuba, Ibaraki, Japan). All mice were housed in the University of Kentucky and Texas A&M University Animal Care Facility, following institutional and National Institutes of Health guidelines. The experiments were approved by the Institutional Animal Care and Use Committees (IACUC) of the University of Kentucky and Texas A&M University. Male mice at 8–12 weeks were used in all experiments.

#### Primary cell cultures

Bone marrow derived macrophages (BMDMs) were prepared as described previously^21^ and seeded into 12-well cell culture plate or 96-well cell culture plate at a density of 1 × 10^6^ cells/well in 1 mL of RPMI-1640 medium containing 15% L929-cell conditioned medium (LCM). BMDMs were allowed to settle overnight and refreshed with Opti-MEM (Life Technologies, Cat#31985–070) before purified protein was added.

Total lung cells were harvested from two-week old mice following the protocol published.^51^ Single cell suspensions were cultured on a 10 cm cell culture dish in DMEM/F12 medium supplemented with antibiotic-antimycotic solution and 10% (v/v) fetal bovine serum (FBS) at 37°C. For monocyte and macrophage depletion in total lung cells, 100 μg/mL clodronate liposome or control liposome (Encapsula NanoSciences, Nashville, TN, Cat # CLD-8909) was added into cells suspensions. When cells reached 90% confluence, cells were then seeded into a 96-well cell culture plate or 12-well cell culture plate at a density of 5×10^5^ cells/mL of culture medium. Cells were allowed to settle overnight and refreshed with 100 μL of Opti-MEM before the indicated proteins were added. All primary cells were confirmed by genotyping and tested negative for mycoplasma contamination.

#### Cell lines

Commercial epithelial and endothelial cells were obtained from Cell Biologics and cultured in the provided medium. Epithelial cells were cultured in basal medium supplemented with 0.1% (v/v) Insulin-Transferrin-Selenium (ITS), 0.1% (v/v) EGF, 2% (v/v) FBS and antibiotic-antimycotic solution. Endothelial cells were cultured in basal medium supplemented with 5% (v/v) FBS, 0.1% (v/v) ECGS, VEGF, EGF, 100 μg/mL heparin and antibiotic-antimycotic solution. Cells were characterized by immunofluorescent staining and tested negative for bacteria, yeast, fungi, and mycoplasma.

### METHOD DETAILS

#### Genetic GSDMD inhibition in fibroblast

B6.Cg-Tg(Col1a2-cre/ERT,-ALPP)7Cpd/2J transgenic mice were crossed with GSDMD floxed mice to generate inducible GSDMD deficient mice in fibroblast. Removal of GSDMD was induced by intraperitoneal injections of tamoxifen (Sigma, T5648) at 100 mg/kg per day for 5 consecutive days at 4 to 5 weeks of age, and subsequent experiments were carried out at 5 weeks post-induction.

#### *In vivo* challenges

For flagellin challenge, purified PA and LFn-flagellin in PBS were administered via retro-orbital injection. For bacterial challenge, mice were injected intraperitoneally with *Salmonella Typhimurium* (ATCC 14028). In the FIP model, fresh stool was dissolved in saline solution to a concentration of 40 mg/mL and administered intraperitoneally to mice, as described previously.^52^

#### *In vitro* challenges

For the study of flagellin induced cytotoxicity, LFn-flagellin with PA were added. When using Nigericin as positive control, cells were primed for 4 h with 1 μg/mL LPS (Sigma, Cat#L4130), then added with 20 μM Nigericin (Invivogen, Cat# tlrl-nig) for 4 h, with or without 5 μM caspase-1 inhibitor Ac-YVAD-cmk (MedChemExpress, Cat#HY-16990).

#### Pharmacological DIC Inhibition

Rat IgG (Sigma) or 1H1 anti-TF antibody (Genentech) at 8 mg/kg was given via retro-orbital injection 2 h prior to PBS or LFn-flagellin/PA injection.

#### Epithelial and endothelial cells isolation

Primary epithelial and endothelial cells were isolated following the protocol published by Messier et al..^51,53^ Single lung cells were incubated with Ep-CAM (Invitrogen, Cat#2083936) or CD102 (BD Pharmingen, Cat#553325) coated dynabeads (Invitrogen, Cat#11035), to isolate epithelial cells or endothelial cells, respectively. Isolated cells were plated in 0.1% gelatin-coated 12-well plate at 37°C in a cell culture incubator with 5% CO_2_. Cells were changed into complete media after 24 h. Culture media was changed every 48 h. Cells were ready for experiments when reached 90% confluent. To study flagellin induced cytotoxicity, cells were seeded into a 96-well cell culture plate at a density of 5×10^5^ cells/mL in complete culture media. Cells were allowed to settle overnight and refreshed with 100 μL of Opti-MEM before the addition of LFn-flagellin/PA.

#### Fibroblast isolation

Fibroblasts were prepared from two-week old mice following the protocol described previously.^54^ Briefly, lungs were minced into very small pieces using a razor blade on a 10 cm tissue culture dish, and then 10 mL of fibroblast culture media (DMEM/F12 containing 10% fetal bovine serum and antibiotic-antimycotic solution) was added and incubated at 37°C for 1–1.5 weeks. Clusters of fibroblasts were visibly growing out of the tissue pieces. When the cells reached 90% confluent, they were seeded into a 96-well cell culture plate at a density of 5×10^5^ cells/mL in the complete culture media. Cells were allowed to settle overnight and refreshed with 100 μL of Opti-MEM before adding LFn-flagellin/PA.

#### Mechanical ventilation

Animals were anesthetized with 2.5% Tribromoethanol, tracheostomized and connected to a Small Animal Ventilator (RWD life science, Cat No. R415). The body temperature was maintained at 35 ± 1 C (via rectal thermometer) using a heating pad. Body fluid homeostasis was maintained by administrating saline (i.p, 100μL/h). Operative care during the period included removal of airway mucus, lubricating the eyes, and rotating the animal. The ventilator was set to an average breathing frequency of 60 breaths/min and a tidal volume of 10 mL/kg with a positive end-expiratory pressure set at 0 cm H_2_O. Oxygen was maintained at 90%.

#### Isolation of lung cells for single-cell RNA sequencing

Mice were divided into two groups, with each group injected with either PBS or LFn-flagellin/PA (3 μg) respectively. After 6 h, mouse lungs were dissected, minced, and placed in gentleMACS C-tubes (Miltenyi biotec, Cat#130-096-334) with 1 mg/mL collagenase A (Worthington Biochemical Corporation, Cat#LS 004196) and 100 μg/mL DNase I (Sigma, Cat#10104159001) in DMEM at 37°C for 30 min. The minced tissues were partially dissociated by running ‘m_lung_01’ on a gentleMACS Dissociator (Miltenyi biotec, Cat#130-093-235), incubated at 37°C for 30 min, and then completely dissociated on a gentleMACS by running ‘m_lung_02’. Cells were washed with 0.1% BSA in PBS, centrifuged at 200 g for 5 min, resuspended in 0.1% BSA in PBS, filtered through a 70-μm strainer. According to its size, the resulting pellet was resuspended in 500–1000 μL of RBC lysis buffer for 1 min until white appearance of the suspension was achieved. The cell suspension was then washed twice and resuspended in PBS containing 0.1% BSA.

#### scRNA-seq library preparation and sequencing

Cell suspensions were assessed for number and viability using the Countess 2.0 automated cell counter (Invitrogen). Three control cell suspensions were pooled to allow for equal cell numbers from each preparation. Similarly, three treatment cell suspensions were pooled for equal numbers of cells from each preparation. Single cell RNA sequencing libraries were generated using the 10x Genomics Next GEM Single Cell 5′ Reagent Kit v2 (dual index). Cells were loaded per manufacturer’s recommendation with a targeted capture of 10,000 cells for each group (control and treatment). Resulting sequencing libraries were quantified via Qubit 2.0 dsDNA assay and checked for quality using the Agilent TapeStation 4200. Equimolar pools were sequenced on the Illumina NovaSeq 6000 2×150 sequencing run. A total of 7,489 cells and 8,230 cells were sequenced for the control and treatment group respectively with approximately 55,000 reads per cell for each group. FASTQ files were uploaded into the 10x Genomics Cloud Analaysis platform to run the Cell Ranger Count v6.1.2 with the Mouse (mm10) 2020-A reference. The output included barcodes, features, and matrix.mtx files, which were subsequently imported into R software for further analysis.

#### scRNA-seq data analysis

All main analyses were performed using Seurat v5.0.3 in R v4.3.6. Quality control was independently performed on each library to find appropriate filtering thresholds for individual libraries (Figures S7A and S7B). For both libraries, only cells with more than 200 detected genes and less than 5% of genes mapped to mitochondrial genes were included. SCTransform was then performed for normalization and selection of highly variable genes using default settings. Seurat objects for each sample were integrated using SelectIntegration-Features(), PrepSCTIntegration(), FindIntegrationAnchors(), and IntegrateData(), with “SCT” as the normalization method. After integration, PCA (default settings), UMAP (dims = 1:40), and clustering (resolution = 0.8) were performed.

To assign cell types to clusters, we assessed canonical markers for major cell types (Epcam: epithelial; Pecam1: endothelial; Ptprc: immune; Col1a1: stromal; Msln: mesothelial)(Hurskainen 2021 Nature Communications) and selected markers genes from the single-cell atlas of mouse lung.^55^ We also performed SingleR against celldex:MouseRNAseqData to assist cell type assignment.^55^

To identify differentially expressed genes in each subset, we used FindAllMarkers(), saved the results, and filtered for significant markers with adjusted *p*-values less than 0.001. Gene name conversion was performed using the bitr() function from the clusterProfiler package, and markers were merged with their corresponding gene IDs. Data preprocessing was done using the separate() function from the tidyr package to separate clusters into cell types and groups. Multiple group enrichment analysis was conducted using the compareCluster() function, and the results were visualized using dotplot() from ggplot2, with different facets for cell types and groups.

We analyzed differential genes between the two groups for macrophages, lung fibroblasts, and endothelial cells, and plotted volcano plots and heatmaps. This comprehensive approach allowed us to elucidate the significant roles of these cell types in the inflammatory response and lung injury under septic conditions.

#### Monocytes/macrophage depletion

Mice were injected retro-orbitally with 40 mg/kg of clodronate liposome or control liposome 24 h before flagellin challenge.^42^

#### LFn fusion protein expression and purification

Proteins were expressed in *E. coli* BL21 strains at 37°C for 4 h with 500 μM IPTG after OD600 reached 0.6–0.8. Bacteria were collected and lysed in 50 mM Tris-HCL and 300 mM NaCl. Proteins containing a His-tag were purified by affinity chromatography using HisPur Ni-NTA resin (Thermo scientific, Cat#88222). Proteins were then eluted with 250 mM imidazole in 50 mM Tris-HCL and 300 mM NaCl, and subsequently dialyzed against PBS to remove imidazole. Protein concentrations were determined by measuring their absorption at 280 nm before sterile filtration.

#### Prothrombin time (PT)

Blood was collected from tribromoethanol (Avertin)-anaesthetized mice by cardiac puncture with a 23-gauge needle attached to a syringe pre-filled with 3.8% trisodium citrate as anticoagulant (final ratio at 1:10). Blood samples were centrifuged at 1,500 g for 15 min at 4°C to obtain plasma. Prothrombin time (PT) was determined with Thromboplastin-D (Pacific Hemostasis, Cat#100357/lot965299) in a manual setting according to manufacturer’s instruction, using CHRONO-LOG #367 plastic cuvette.^21^

#### Plasma TAT concentrations

Plasma TAT concentrations were determined using a mouse TAT ELISA kit (Abcam, Cat#ab137994) at 1:50 dilution according to manufacturer’s instruction. Plasma was collected as mentioned above for PT.

#### Determination of pulmonary microvascular permeability

Lungs were isolated 1 h after injection of flagellin and the microvessel capillary filtration coefficient (Kfc) was measured as described previously.^31^ In brief, after 20-min equilibration perfusion to establish an isogravimetric condition, the outflow pressure was rapidly elevated by 10 cm of H_2_O for 20 min. Lung preparations gained weight in response to the pressure increase, reflecting the net fluid accumulation. Lungs were dissected free of nonpulmonary tissue, and lung dry weight was determined. Kfc was calculated from the slope of the weight change normalized to the pressure change and lung dry weight.

#### Pulse oximetry

Each mouse was anesthetized using 2.5% Tribromoethanol to facilitate placement of a PawClip Sensor and allowed to acclimatize for 5 min. Arterial O_2_ saturation level was measured continuously using PhysioSuite (Kent Scientific, Cat#13-005-112) in accordance with manufacturer’s instructions. Measurements were recorded every 5 min after flagellin injection, up to 60 min. This was average for all parameters.

#### IL-1R blocking

C57BL6 mice were injected intravenously with 1 mg/kg of recombinant human IL-1 receptor antagonist (IL-1RA, PeproTech, Cat# 200–01RA) 10 min before the injection with LFn-flagellin/PA.

#### Bronchoalveolar lavage (BAL) and lung histology

BAL was collected in the lungs of mice after flagellin treatment. The trachea was exposed and cannulated to infuse the lung with 0.7 mL PBS/2 mM EDTA twice. BAL was collected, centrifuged, and stored on ice. The total macrophages numbers in the BAL were determined using a Neubauer chamber. Mice were perfused via both right and left ventricles with PBS and then perfusion-fixed with 10% formalin under physiological pressure for 30–45 min. The right lung of each mouse was collected and embedded in paraffin, then sectioned serially at 5 μm for H&E staining.

#### ELISA analysis

IL-6, IL-1β, and TNF-α levels in the BAL were measured using Invitrogen ELISA kits (Thermo Fisher Scientific) according to the manufacturer’s instructions.

#### Cytotoxicity

Pyroptotic cell death was measured by the lactate dehydrogenase (LDH) CytoTox 96 Non-Radioactive Cytotoxicity Assay kit (Promega, Cat#G1780) according to manufacturer’s instruction.

#### Western blotting

To assay caspase-1 activation and IL-1β cleavage by western blotting, supernatants from cells treated with indicated stimuli were subjected to trichloroacetic acid (TCA) precipitation. Proteins in the precipitates were analyzed by immunoblotting. Cell lysates were blotted with antibodies against caspase-1, IL-1β, caspase-8, GSDMD, NLRC4, ASC and actin, respectively. Both pro-caspase-1 and p20 caspase-1 were determined using anti-caspase-1 (p20) (Adipogen, Cat#AG-20B-0042) at 1:1000 dilution. Pro-IL-1β and IL-1β (p17) were detected using anti-IL-1β (GeneTex Cat#GTX74034) at 1:1000 dilution. Caspase-8 (p18) was detected using anti-caspase-8 (Cell Signaling Technology, Cat#8592) at 1:1000 dilution. GSDMD was detected using anti-GSDMD (Santa cruz biotechnology, Cat#393582) at 1:500 dilution. NLRC4 was detected using anti-NLRC4 (Abcam, ab201792) at 1:1000 dilution. ASC was detected using anti-ASC (Cell Signaling Technology, Cat#67824) at 1:1000 dilution. Actin was detected using anti-actin (Cell Signaling Technology, Cat#4970) at 1:1000 dilution. Blots were imaged using BIO-RAD ChemiDoc MP imaging system and Azure Imaging Systems.

#### Flow cytometry

Anti-CD45-APC-Cy7, clone 30-F11 (BD Biosciences, catalog no. 557659); And anti-F4/80-FITC, clone BM8 (BioLegend, catalog no. 123107), PI (BioLegend, catalog no. 421301) were used for flow cytometric analysis in the study. Macrophages were identified as CD45^+^F4/80^+^. Data were acquired on a Cytek Aurora (Cytek Biosciences) and analyzed with FlowJo version 10.07. To obtain single cell suspension, lung tissues were digested with a cocktail of 1 mg/mL collagenase A (Worthington Biochemical, catalog no. LS 004196) and 100 μg/mL DNase I (Sigma, catalog no. 10104159001) in DMEM at 37°C for 30 min.

### QUANTIFICATION AND STATISTICAL ANALYSIS

Statistical analysis was performed in Prism GraphPad 6.0.0. Data are represented as mean ± SEM. Student’s t test (two-sided) was used to compare two-group data with normal distribution and equivalent variance; ANOVA with Holm-Sidak multiple comparisons were used for multiple groups, and two-way ANOVA repeated measures with Holm-Sidak multiple comparisons were used for time point studies. A *p* value < 0.05 was considered significant.

### ADDITIONAL RESOURCES

No new resources or clinical trials were generated in this study.

## Supplementary Material

1

## Figures and Tables

**Table:** KEY RESOURCES TABLE

REAGENT or RESOURCE	SOURCE	IDENTIFIER
Antibodies		
1H1 anti-TF antibody	Genentech; Daniel Kirchhofer; Wu et al.^21^	N/A
Anti-caspase-1(p20) (mouse)	Adipogen	Cat#AG-20B-0042-C100 RRID:AB_2755041
Anti-IL1β	GeneTex	Cat#GTX74034
Anti-β-Actin	Cell Signaling Technology	Cat# 4970; RRID:AB_2223172
Anti-GSDMD	Santa cruz biotechnology	Cat# 393582
Anti-caspase-8 (p18) (mouse)	Cell Signaling Technology	Cat#8592; RRID:AB_10891784
Anti-CD45-APC-Cy7, clone 30-F11	BD Biosciences	Cat#557659; RRID:AB_396774
Anti-F4/80-FITC, clone BM8	BioLegend	Cat#123107; RRID:AB_893500
Anti-Ep-CAM	Invitrogen	Cat#2083936
Anti-CD102	BD Biosciences	Cat#553325; RRID:AB_394783
Anti-NLRC4	Abcam	Cat#ab201792
Anti-ASC	Cell Signaling Technology	Cat#67824; RRID:AB_2799736
Bacterial and virus strains		
Salmonella typhimurium	ATCC	14028
Biological samples		
Ctrl Wild Type mice lung sample for single-cell RNA-seq, 1 replicate	This paper	N/A
Flagellin/PA treated Wild Type mice lung sample for single-cell RNA-seq, 1 replicate	This paper	N/A
Chemicals, peptides, and recombinant proteins		
PA	This paper	N/A
LFn-EprJ	This paper	N/A
LFn-Flagellin	This paper	N/A
LFn-Flagellin-3A	This paper	N/A
LPS (E. coli O111:B4)	Sigma	Cat#L4130
Tamoxifen	Sigma	T5648
Opti-MEM	Life Technologies	Cat#31985-070
Clodronate liposome kit	Encapsula NanoSciences	Cat# CLD-8909
Nigericin	Invivogen	Cat# tlrl-nig
Ac-YVAD-cmk	MedChemExpress	Cat#HY-16990
Collagenase A	Worthington Biochemical Corporation	Cat#LS 004196
DNase I	Sigma	Cat#10104159001
IL-1RA	PeproTech	Cat# 200-01RA
Propidium Iodide	BioLegend	Cat#421301
HisPur Ni-NTA resin	ThermoFisher	Cat#88222
Critical commercial assays		
Thromboplastin-D	Pacific Hemostasis	Cat#100357
TAT ELISA kit	Abcam	Cat#ab137994
CytoTox 96 Non-Radioactive Cytotoxicity Assay	Promega	Cat#G1780
Plastic cuvette	CHRONO-LOG	Cat#367
IL-6 ELISA kit	ThermoFisher Scientific	Cat#88-7064-88
IL-1β ELISA kit	ThermoFisher Scientific	Cat#88-7013A-88
TNF-α ELISA kit	ThermoFisher Scientific	Cat#88-7324-88
Deposited data		
Raw data for single-cell RNA-seq	This paper	GEO:GSE269196
Experimental models: Cell lines		
Mouse Primary Bone Marrow Derived Macrophages	This paper	N/A
Mouse Lung Epithelial Cells	This paper	N/A
Mouse Lung Endothelial Cells	This paper	N/A
Mouse Lung Fibroblast Cells	This paper	N/A
Mouse Primary Alveolar Epithelial Cells	Cell Biologics	Cat#C57-6053
Mouse Primary Lung Microvascular Endothelial Cells	Cell Biologics	Cat#C57-6011
Experimental models: Organisms/strains		
C57BL/6J	The Jackson Laboratory	JAX:000664; RRID:IMSR_JAX:000664
*Casp1* ^*−/−*^	Genentech Inc; Nobuhiko Kayagaki; Kayagaki et al.^47^	N/A
*Gsdmd* ^*−/−*^	National Institute of Genetics, Japan; Toshihiko Shiroishi; Wu et al.^21^	N/A
*Gsdmd* ^fl/fl^	National Institute of Genetics, Japan; Toshihiko Shiroishi; Wu et al.^21^	N A
*NAIP* ^*−/−*^	The Jackson Laboratory	JAX:032660; RRID:IMSR_JAX:032660
*Gsdme* ^*−/−*^	The Jackson Laboratory	JAX:032411; RRID:IMSR_JAX:032411
*Tlr5* ^*−/−*^	The Jackson Laboratory	JAX:008377; RRID:IMSR_JAX:008377
B6.Cg-Tg(Col1a2-cre/ERT,-ALPP) 7Cpd/2J	The Jackson Laboratory	JAX:029567; RRID:IMSR_JAX:029567
*CaspS* ^*−/−*^ */Ripk3* ^*−/−*^	Genentech Inc; Nobuhiko Kayagaki; Lee et al.^45^	N/A
*Nlrc4* ^*−/−*^	Genentech Inc; Lee et al.^45^	N/A
Software and algorithms		
FlowJo v10.07	FLOWJO	https://www.flowjo.com/learn/flowjo-university/flowjo/getting-started-in-flowjo/131
GraphPad Prism 6	GraphPad	https://www.graphpad.com/scientific-software/prism/
R Studio v. 4.3.6	RStudio	https://cran.r-project.org/
Other		
Small Animal Ventilator	RWD life science	Cat#No. R415
GentleMACS C-tubes	Miltenyi biotec	Cat#130-096-334
GentleMACS Dissociator	Miltenyi biotec	Cat#130-093-235
PhysioSuite	Kent Scientific	Cat#13-005-112

**Table 1.** Percentage of each cell cluster before and after flagellin treatment

Group	Cell type	Frequency	Total	%
Ctrl	neutrophils	217	4,731	0.04586768
Treat	neutrophils	1,204	6,539	0.18412601
Ctrl	CD4^+^ T cells	491	4,731	0.10378356
Treat	CD4^+^ T cells	851	6,539	0.13014222
Ctrl	endothelial cells	1,357	4,731	0.28683154
Treat	endothelial cells	1,543	6,539	0.2359688
Ctrl	B cells	776	4,731	0.16402452
Treat	B cells	611	6,539	0.09343936
Ctrl	CD8^+^ T cells	386	4,731	0.08158952
Treat	CD8^+^ T cells	552	6,539	0.08441658
Ctrl	interstitial fibroblasts	486	4,731	0.1027267
Treat	interstitial fibroblasts	616	6,539	0.09420401
Ctrl	epithelial cells	217	4,731	0.04586768
Treat	epithelial cells	417	6,539	0.06377122
Ctrl	macrophages	256	4,731	0.05411118
Treat	macrophages	112	6,539	0.017128
Ctrl	lipofibroblasts	114	4,731	0.02409639
Treat	lipofibroblasts	124	6,539	0.01896314
Ctrl	NK cells	203	4,731	0.04290848
Treat	NK cells	209	6,539	0.03196207
Ctrl	Naive T cells	34	4,731	0.00718664
Treat	Naive T cells	116	6,539	0.01773972
Ctrl	myofibroblast	106	4,731	0.02240541
Treat	myofibroblast	99	6,539	0.01513993
Ctrl	platelets	30	4,731	0.00634115
Treat	platelets	42	6,539	0.006423
Ctrl	megakaryocytes	58	4,731	0.01225956
Treat	megakaryocytes	43	6,539	0.00657593

Ctrl, control; NK, natural killer.

## Highlights

- Activation of the NAIP/NLRC4 inflammasome triggers acute lung injury in mice
- NAIP/NLRC4 inflammasome components are expressed in macrophages and pulmonary fibroblasts
- Acute lung injury caused by NAIP/NLRC4 inflammasome activation is dependent on pyroptosis
- Pyroptosis of both pulmonary fibroblasts and macrophages contributes to lung injury
