# Supplementary material for: Pyroptosis of pulmonary fibroblasts and macrophages through NLRC4 inflammasome leads to acute respiratory failure
Source: Cell Rep. Author manuscript; Available in PMC 2025 May 19. (PMC12087274; doi:10.1016/j.celrep.2025.115479)
Supplement: 1 [file NIHMS2076714-supplement-1.pdf]

Cell Reports, Volume *44*

Supplemental information

Pyroptosis of pulmonary fibroblasts and macrophages through NLRC4 inflammasome leads to acute respiratory failure

Yan Zhang, Guoying Zhang, Brittany Dong, Ankit Pandeya, Jian Cui, Samuel dos Santos Valenca, Ling Yang, Jiaqian Qi, Zhuodong Chai, Congqing Wu, Daniel Kirchhofer, Toshihiko Shiroishi, Fadi Khasawneh, Min Tao, Feng Shao, Christopher M. Waters, Yinan Wei, and Zhenyu Li

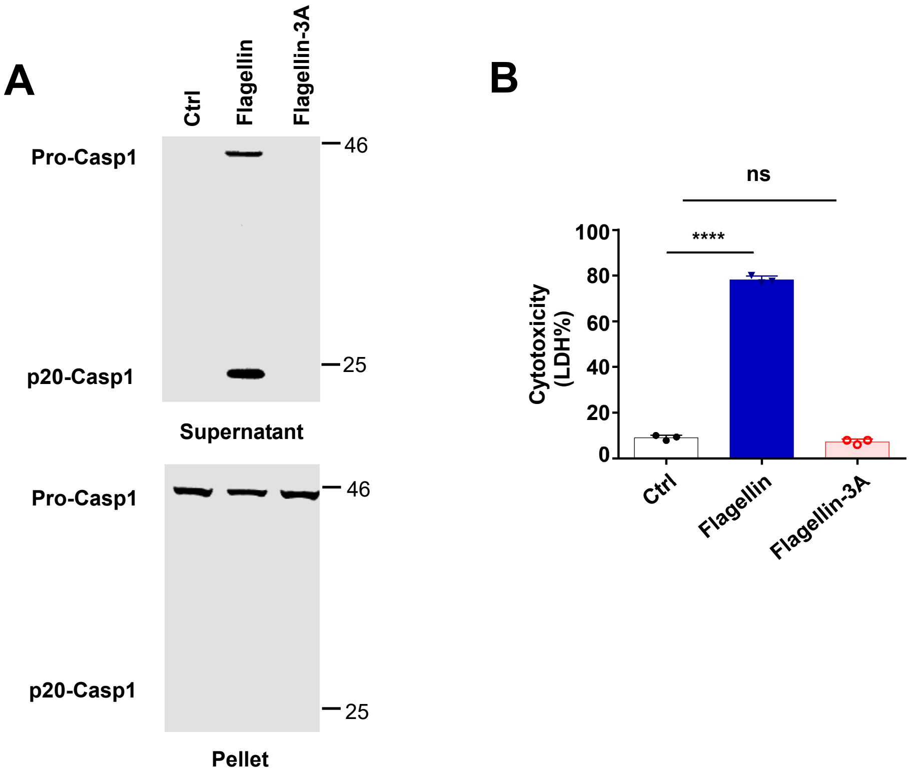

Figure S1. Flagellin induces inflammasome activation and pyroptosis in mouse BMDMs, Related to Figure 1

(A and B) BMDMs from C57BL/6J mice were incubated with PA (Ctrl), 1 μg/mL LFn-flagellin/PA, or 1 μg/mL LFn-flagellin-3A mutant (inactive form) plus PA for 90 minutes. Pro and p20 caspase-1 in the supernatant and lysate were detected by Immunoblot (A). Supernatants from cell cultures were precipitated to concentrate protein prior to Immunoblot. Cell viability was determined by quantitation of LDH as an indicator of cytotoxicity (B). Data are representative of 3 biological replicates. Error bars denote SEM. n=3, **** $p < 0.01$ versus Ctrl (one-way ANOVA with Holm-Sidak multiple comparisons).

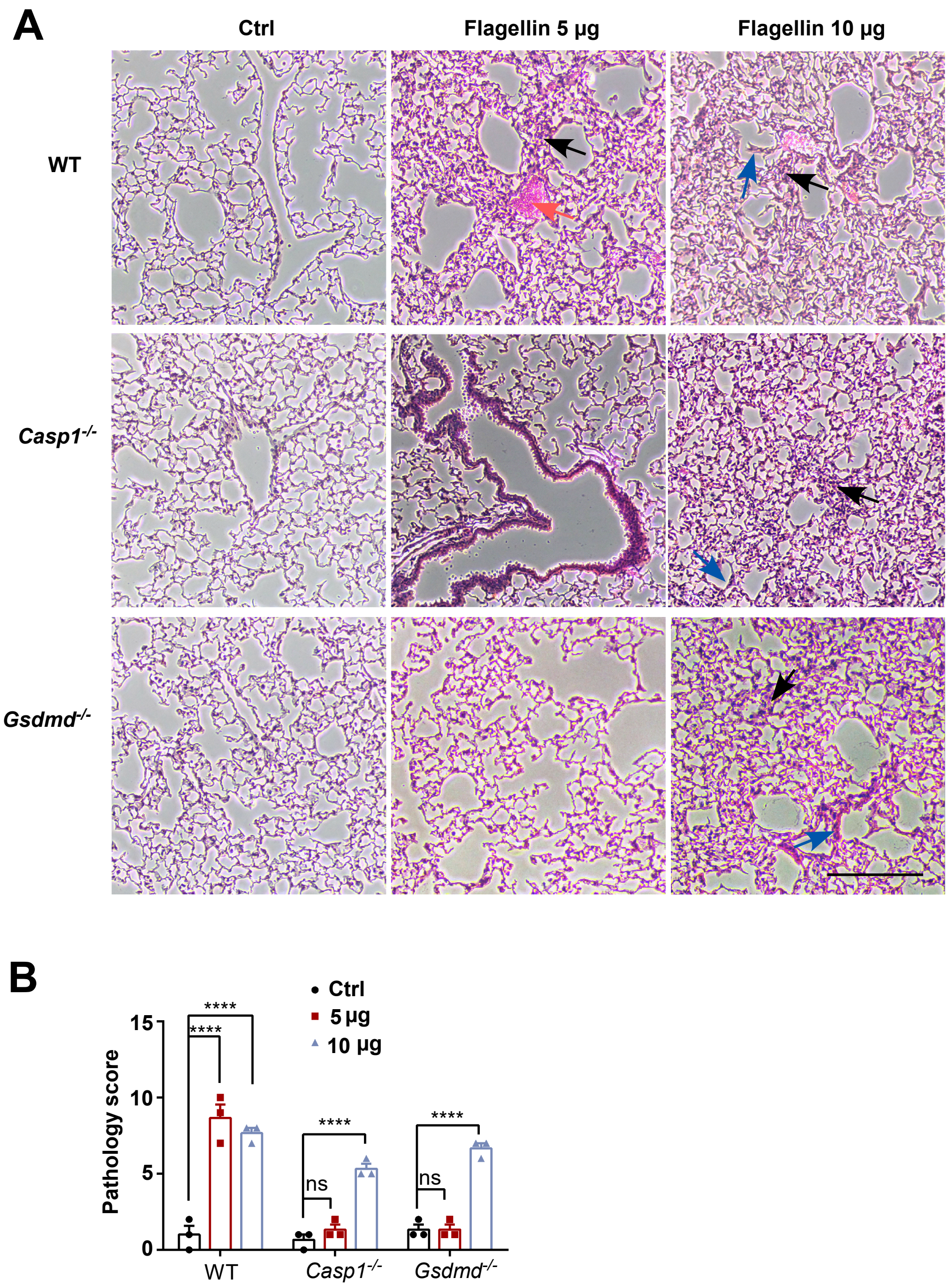

Figure S2. Flagellin induces lung inflammation in mice, Related to Figures 1,2,5

Histological evaluation of lung morphology after challenge with flagellin. C57BL/6J (WT), *Casp1*$^{-/-}$ and *Gsdmd*$^{-/-}$ mice were injected intravenously with different dose of LFn-flagellin plus PA. After indicated time, mice were euthanized and perfused with PBS and then perfusion fixed with 10% formalin under physiological pressure. H&E stained lung sections obtained from WT, *Casp1*$^{-/-}$ and *Gsdmd*$^{-/-}$ mice were shown (A). Pathology score were shown on the bottom (B).Note: Black arrow points to mononuclear cells infiltration in lungs. Blue arrow to thickened alveolar wall. Red arrow to hemorrhage. Scale bars denote 200 µm. Data are representative of 3 independent experiments (biological replicates). ns, $p > 0.05$. ****$p < 0.01$ versus Ctrl (two-way ANOVA with Holm-Sidak multiple comparisons).

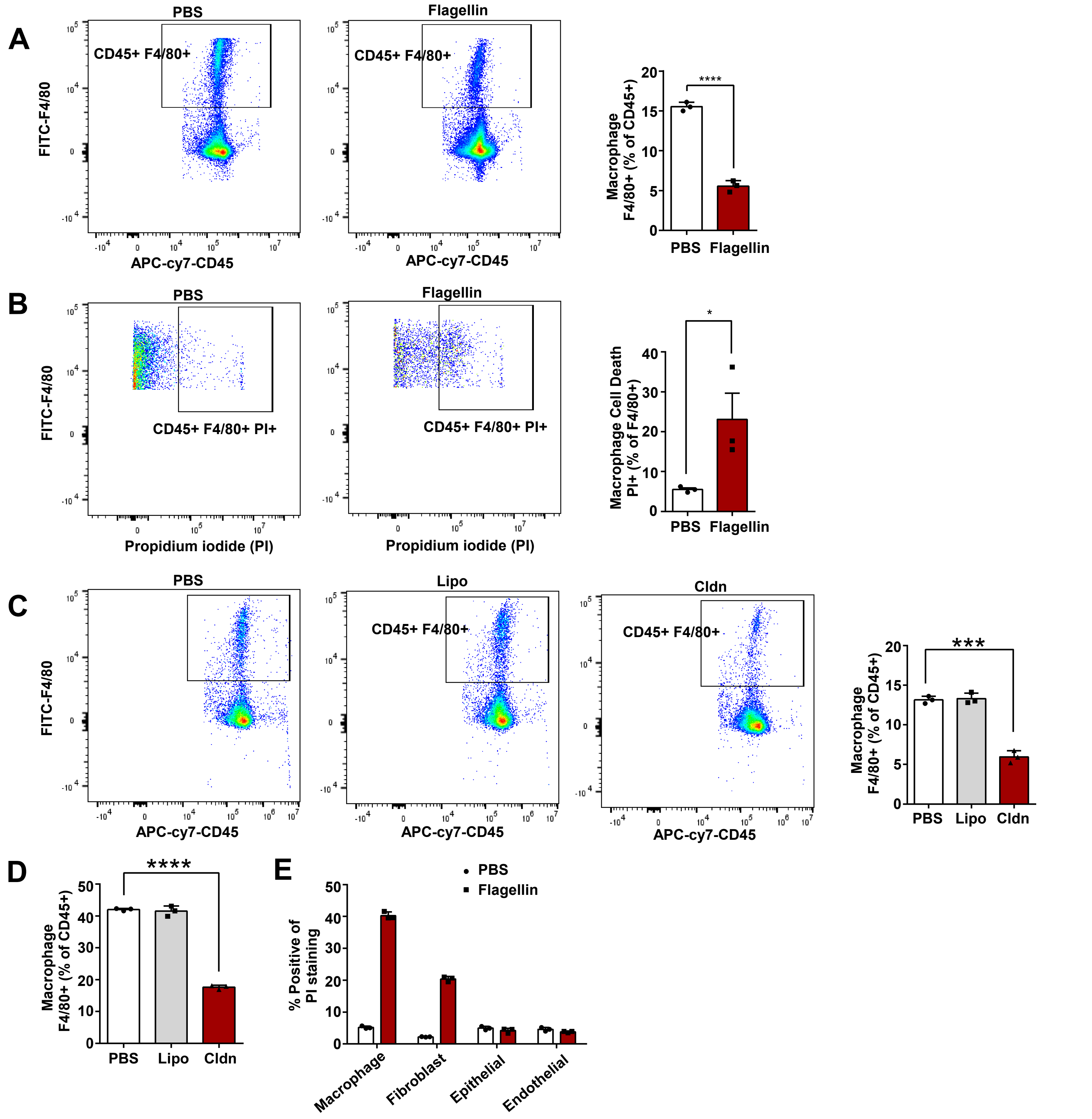

Figure S3. Reduction of lung macrophages following liposome clodronate or flagellin treatment, Related to Figures 3,4,5

(A and B) Flagellin induces macrophage cell death in the lung in vivo. Mice were challenged with PBS (Ctrl) or Flagellin (3 μg PA plus LFn-Flagellin per mouse). Lung tissues were collected 90 minutes after injection. Representative flow cytometric analysis of macrophage (F4/80+) population is shown on the left, the corresponding quantification is shown on the right (B). Propidium iodide (PI) was used to evaluate macrophage cell viability. Error bars denote SEM.n=3, * $p < 0.05$ versus Ctrl (Student's t-test).

(C) Liposome clodronate significantly reduces lung macrophages in vivo.Representative flow cytometric analysis of macrophage (F4/80+) population from lung is shown on the left, the corresponding quantification is shown on the right. Error bars denote SEM.n=3, *** $p < 0.01$ versus Ctrl (one-way ANOVA with Holm-Sidak multiple comparisons).

(D) Liposome clodronate significantly reduces lung macrophages in vitro. Error bars denote SEM. n=3, **** $p < 0.001$ versus Ctrl (one-way ANOVA with Holm-Sidak multiple comparisons).

(E) Flagellin induces cell death in cultured pan-lung macrophages and fibroblasts, but not in epithelial or endothelial cells, assessed by flow cytometric analysis. PI was used to evaluate cell viability.

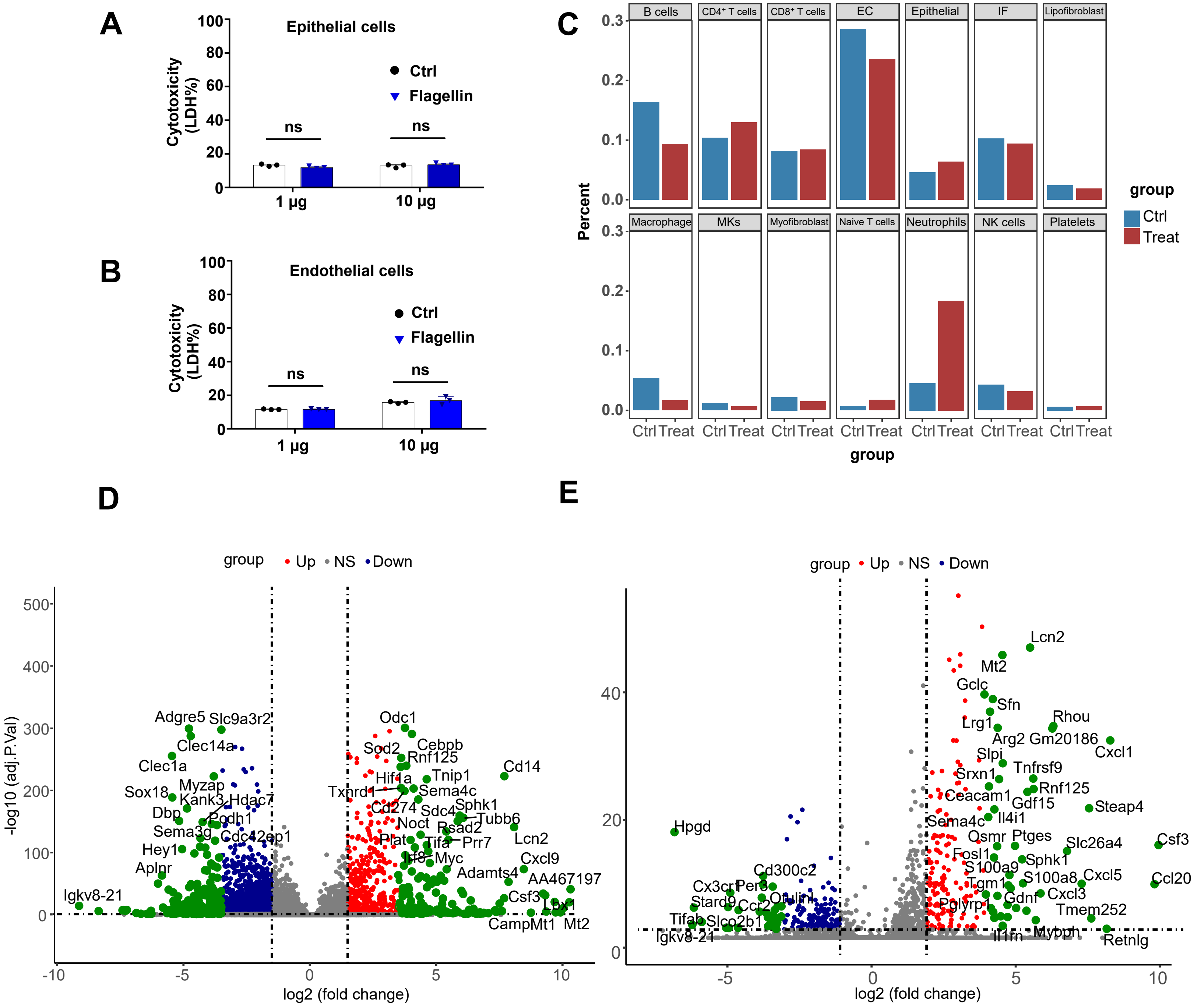

Figure S4. Effects of flagellin treatment on cell death and single-cell RNA sequencing analysis of cell populations, Related to Figures 3, 4

(A and B) Commercial lung epithelial (A) and endothelial (B) cells from Cell Biologics were incubated with PA (Ctrl), 1 µg/mL LFn-flagellin/PA, or 10 µg/mLLFn-flagellin/PA, for 6 hours. Supernatant from cell cultures were used to measure LDH concentration, indicating cytotoxicity. Data are representative of 3 biological replicates. Error bars denote SEM; n=3, ns, $p > 0.05$ versus Ctrl (two-way ANOVA with Holm-Sidak multiple comparisons).

(C) The proportions of different cell populations before and after flagellin treatment. EC: Endothelial cell. MKs: Megakaryocytes. IF: Interstitial fibroblasts.

(D and E) Volcano plot showing significant changes in genes expression in the endothelial cell population (D) and epithelial cell population (E) following flagellin treatment.

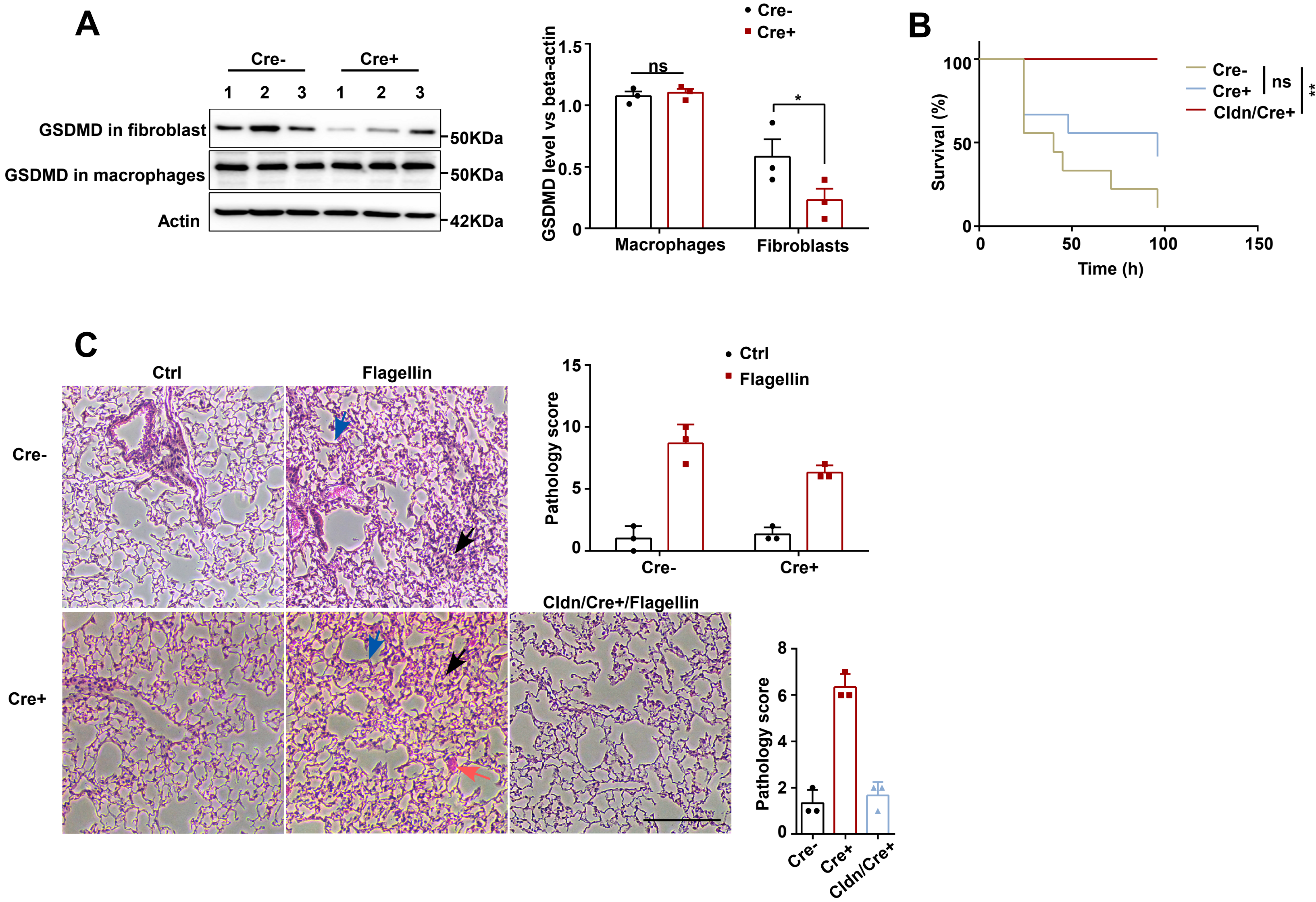

Figure S5. Role of GSDMD in fibroblast pyroptosis and lung injury induced by flagellin, Related to Figure 4

(A) Western blotting was performed to confirm GSDMD deletion exclusively in fibroblast, but not in macrophages. Quantification was shown on the right.*p < 0.05, ns, not significant.

(B) Kaplan-Meier survival plots of *Gsdmd*$^{fl/fl}$/*Col1a2 Cre*$^{-}$ or *Gsdmd*$^{fl/fl}$/*Col1a2 Cre*$^{+}$ mice injected with 5 µg LFn-flagellin/PA. Cre+ mice were further treated with Cldn 24 hours prior to flagellin injection. **p < 0.01,ns, not significant.

(C) Histological evaluation of lung morphology after challenge with flagellin. *Gsdmd*$^{fl/fl}$/*Col1a2 Cre*$^{-}$ or *Gsdmd*$^{fl/fl}$/*Col1a2 Cre*$^{+}$ mice injected with 5 µg LFn-flagellin/PA. Cre+ mice were further treated with Cldn 24 hours prior to flagellin injection (Note: Black arrow points to mononuclear cells infiltration in lungs. Blue arrow to thickened alveolar wall. Red arrow to hemorrhage. Scale bars denote 200 µm. Data are representative of 3 independent experiments (biological replicates).

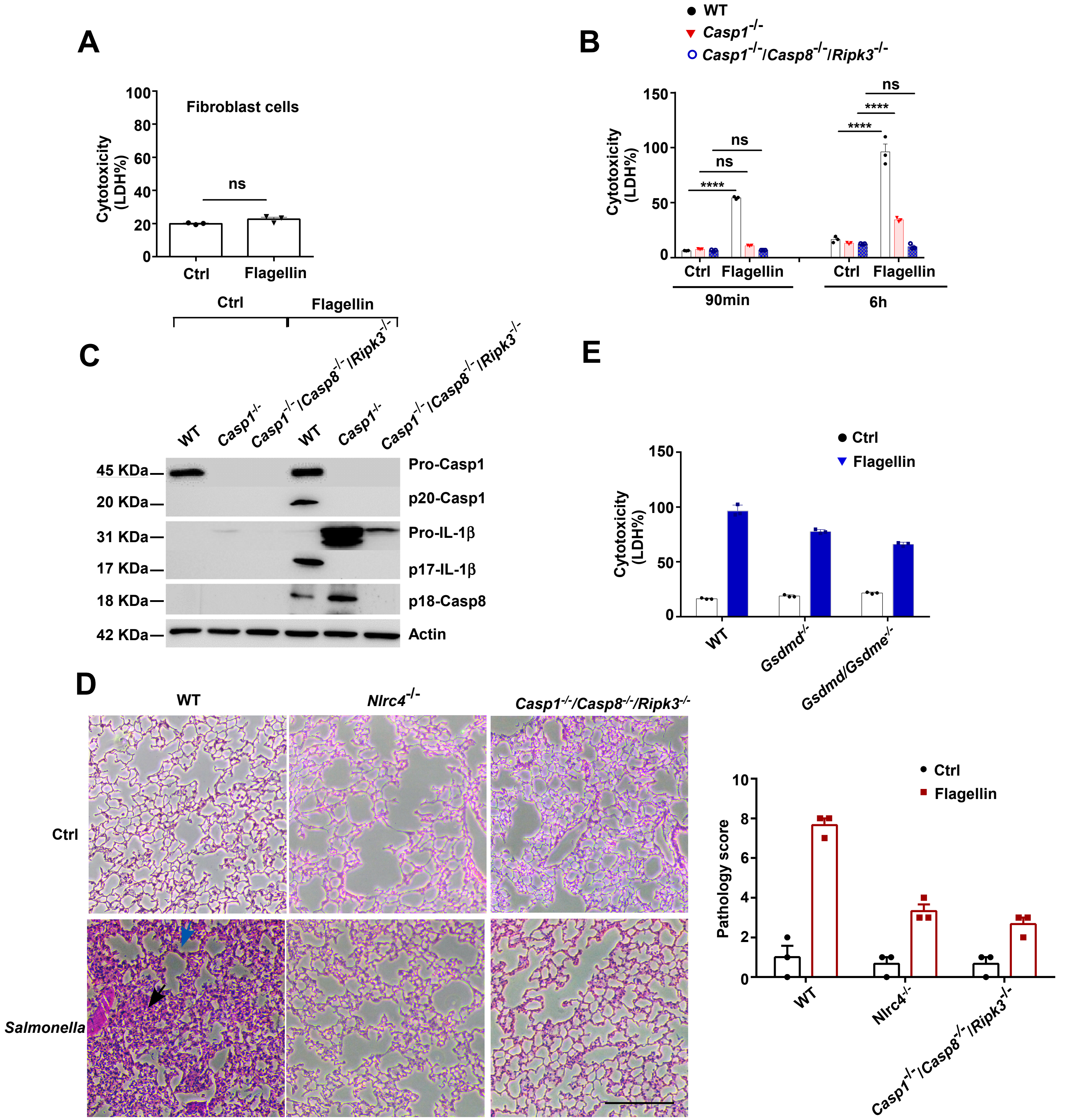

Figure S6. Flagellin induced cell death: caspase and gasdermin pathways across cell types, Related to Figures 5 and 6

(A) Lung fibroblasts isolated from *Casp1*$^{-/-}$ mice were incubated with PA (Ctrl),10 μg/mL LFn-flagellin/PA for 6 hours. (Error bars denote SEM; n=3, ns, $p > 0.05$ versus Ctrl (two-tailed unpaired t test).

(B and C) BMDMs from WT, *Casp1*$^{-/-}$ and *Casp1*$^{-/-}$*/Casp8*$^{-/-}$*/RIPK3*$^{-/-}$ mice were incubated with 1 μg/mL LFn-flagellin/PA for 90 min and 6 hours, respectively. LDH release in supernatants were measured (B). n = 3. Data is represented as mean ± SEM. ns, $p > 0.05$. ****$p < 0.01$ versus Ctrl (two-way ANOVA with Holm-Sidak multiple comparisons). Caspase-1, IL-1β and caspase-8 in cell lysate and supernatant were detected by Western blot (C).

(D) Histological evaluation of lung morphology after challenge with *Salmonella.* WT, *Nlrc4*$^{-/-}$, and *Casp1*$^{-/-}$*/Casp8*$^{-/-}$*/Ripk3*$^{-/-}$ mice were injected intraperitoneally with 2*10^^{8} cfu *Salmonella Typhimurium* for 6 hours. Note: Black arrow points to mononuclear cells infiltration in lungs. Blue arrow to thickened alveolar wall. Scale bars denote 200 μm. Data are representative of 3 independent experiments (biological replicates).

(E) BMDMs from WT, *Gsdmd*$^{-/-}$ and *Gsdmd*$^{-/-}$*/Gsdme*$^{-/-}$ mice were incubated with 1μg/mL LFn-flagellin/PA for 90 min. LDH release in supernatants were measured. n = 3.

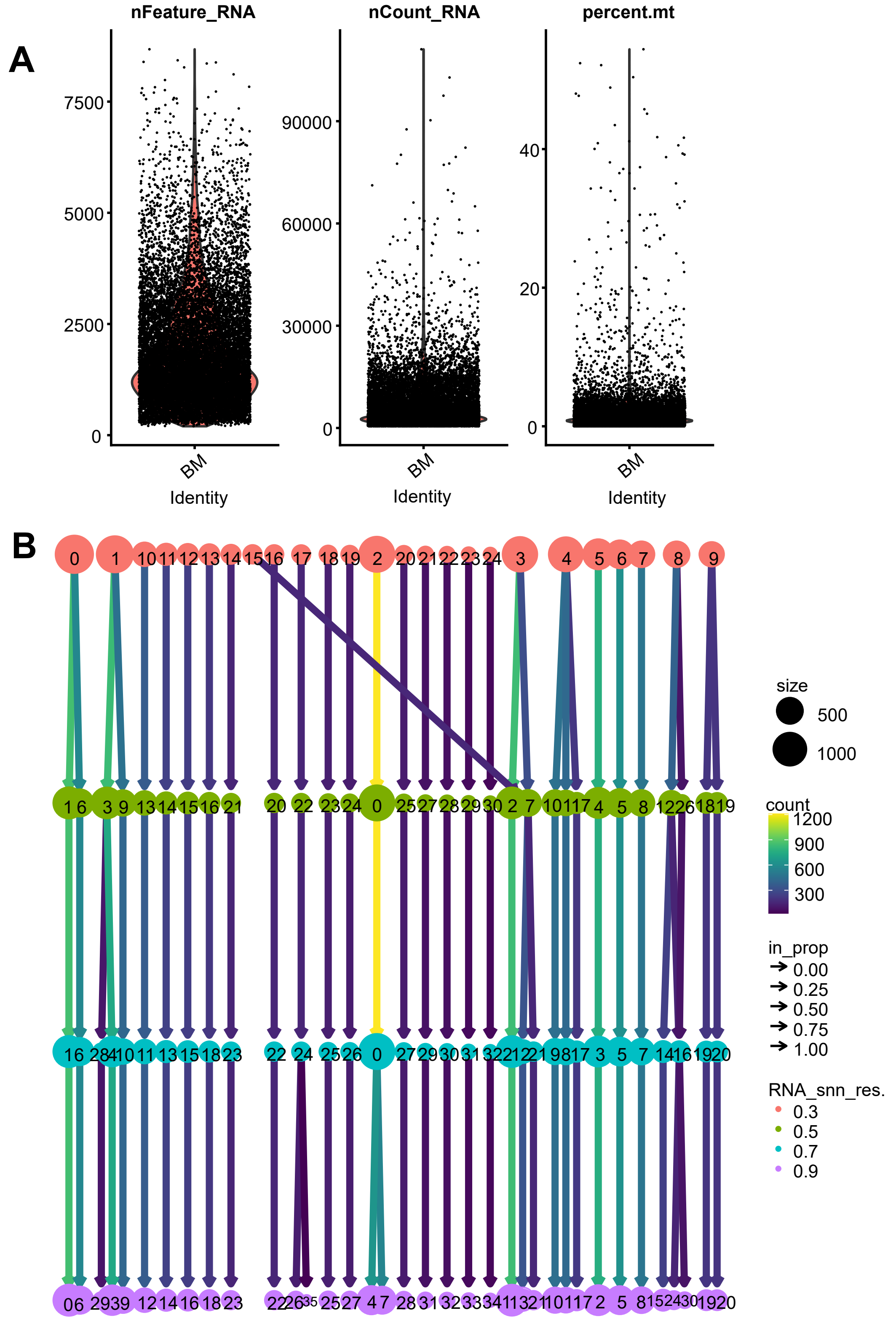

Figure S7. Quality control of the single-cell RNA-seq data, Related to Figure 4

(A) The dot plot shows that the feature RNA and total RNA capture numbers are within a reasonable range, and most mitochondrial RNA is within 5%, indicating good cell viability.

(B) PCA (Principal Component Analysis) was performed for dimensionality reduction, dividing all cells into various groups. 30 clusters were identified at a resolution of 0.5.
